# BRG1-SWI/SNF-dependent regulation of the *Wt1* transcriptional landscape mediates epicardial activity during heart development and disease

**DOI:** 10.1038/ncomms16034

**Published:** 2017-07-24

**Authors:** Joaquim Miguel Vieira, Sara Howard, Cristina Villa del Campo, Sveva Bollini, Karina N. Dubé, Megan Masters, Damien N. Barnette, Mala Rohling, Xin Sun, Laura E. Hankins, Daria Gavriouchkina, Ruth Williams, Daniel Metzger, Pierre Chambon, Tatjana Sauka-Spengler, Benjamin Davies, Paul R. Riley

**Affiliations:** 1 Burdon Sanderson Cardiac Science Centre, Department of Physiology, Anatomy and Genetics, University of Oxford, Oxford OX1 3PT, UK; 2 Molecular Medicine Unit, UCL Institute of Child Health, London WC1N 1EH, UK; 3 Weatherall Institute of Molecular Medicine, John Radcliffe Hospital, University of Oxford, Oxford OX3 9DS, UK; 4 Institut de Génétique et de Biologie Moléculaire et Cellulaire, INSERM U964/CNRS UMR 7104/Université de Strasbourg, 67404 IllKirch Cedex, France; 5 Wellcome Trust Centre for Human Genetics, University of Oxford, Oxford OX3 7BN, UK

## Abstract

Epicardium-derived cells (EPDCs) contribute cardiovascular cell types during development and in adulthood respond to Thymosin β4 (Tβ4) and myocardial infarction (MI) by reactivating a fetal gene programme to promote neovascularization and cardiomyogenesis. The mechanism for epicardial gene (re-)activation remains elusive. Here we reveal that BRG1, the essential ATPase subunit of the SWI/SNF chromatin–remodelling complex, is required for expression of Wilms’ tumour 1 (*Wt1*), fetal EPDC activation and subsequent differentiation into coronary smooth muscle, and restores *Wt1* activity upon MI. BRG1 physically interacts with Tβ4 and is recruited by CCAAT/enhancer-binding protein β (C/EBPβ) to discrete regulatory elements in the *Wt1* locus. BRG1-Tβ4 co-operative binding promotes optimal transcription of *Wt1* as the master regulator of embryonic EPDCs. Moreover, chromatin immunoprecipitation-sequencing reveals BRG1 binding at further key loci suggesting SWI/SNF activity across the fetal epicardial gene programme. These findings reveal essential functions for chromatin–remodelling in the activation of EPDCs during cardiovascular development and repair.

## Abstract (web summary)

Priming of the adult mouse heart with Tβ4 activates dormant epicardium-derived cells to aid repair of injured myocardium. Here, Vieira *et al*. explain this process and show that Tβ4 binds a chromatin remodeller BRG1 and activates Wt1, the key regulator of epicardial epithelial-to-mesenchymal transformation, by altering the epigenetic landscape of the Wt1 locus.

SWItch/Sucrose NonFermentable (SWI/SNF) complexes comprise 11–15 protein subunits and utilize the energy from adenosine triphosphate (ATP) hydrolysis to slide or evict nucleosomes^1^. By reshaping the nucleosome landscape SWI/SNF complexes may also lead to repression of their target genes^2^. BRG1 (Brahma-related gene 1; gene alias *SMARCA4*) is the ATPase catalytic subunit of SWI/SNF, although mammals may also utilize BRM (Brahma; gene alias *SMARCA2*) as an alternative catalyst to remodel targeted chromatin^2^,^3^. BRG1 contains one bromodomain that recognizes acetylated lysines in histone tails, facilitating SWI/SNF binding in active enhancers and near the transcriptional start site (TSS) and, subsequently, enabling RNA polymerase II (RNAPII) occupancy and gene transcription^1^. BRG1 is essential for embryonic development, while BRM seems to be dispensable due to BRG1 compensation^4^,^5^,^6^. In the heart, BRG1 is required for endocardium and myocardium formation^7^,^8^,^9^, whereas BRM functionally compensates for BRG1 in the maintenance of adult coronary vascular endothelial cells^10^. Of significance, the subunit BAF180 (BRG1-associated factor 180, also known as polybromo protein 1) specifically forms SWI/SNF complexes containing BRG1 (PBAF remodelling complexes) and is required for correct cardiac chamber maturation and epicardial epithelial-to-mesenchymal transition (EMT). Mice deficient in BAF180 phenocopy cardiac knockdown of the monomeric-actin binding peptide Tβ4 (refs 2, 11, 12, 13).

The epicardium is the outermost layer of the heart and has been proposed as a source of cardiovascular regeneration in heart disease^14^. During embryogenesis EPDCs play an essential role contributing interstitial fibroblasts, smooth muscle, endothelial cells and potentially cardiomyocytes to the growing heart^15^,^16^,^17^. Different molecular markers have been employed to follow the fate of EPDCs including the transcription factors Wt1, Tbx18 (T-box factor 18) and Tcf21 (transcription factor 21), and the retinoic acid synthetizing enzyme Raldh2 (ref. 18). Wt1 is a zinc finger-containing transcription factor which functions as the master regulator of epicardial EMT, a process required for myocardial growth and coronary vasculature formation^19^,^20^. In this regard, Wt1 sits atop of a hierarchy of embryonic epicardial gene loci and directly controls the transcription of *SnaiI* (*Snail 1*) and *CdhI* (epithelial cadherin; *e-cadherin*)^19^, and regulates β-catenin and RALDH2 signalling pathways as required during epicardial EMT^21^. The activity of these key developmental genes is gradually lost during pregnancy, leading to epicardial quiescence in the postnatal heart, however, *Wt1* is up-regulated in the adult epicardium after MI, an event associated with EPDC mobilization and differentiation into a default fibroblast fate^18^,^22^. Pre-treatment with Tβ4 significantly enhances this epicardial response, promoting neovascularization and cardiomyogenesis in the adult injured heart^23^,^24^. Even though events downstream of Wt1 function have been well described, the upstream regulation of *Wt1* activity during either development or adult heart injury remains unknown.

Here we report an epigenetic mechanism which activates the transcriptional landscape of *Wt1* promoting epicardial activity in the developing and adult heart after MI. Specifically, we identify a novel BRG1–SWI/SNF function, implicating chromatin–remodelling of the *Wt1* locus as a critical event in determining the activation and fate of embryonic EPDCs. Tβ4 physically interacts with BRG1 promoting optimal *Wt1* re-expression in the adult epicardium after MI. The pioneer factor C/EBPβ co-localizes with SWI/SNF complexes to evolutionary conserved regions (ECRs) of the *Wt1* locus located proximal to the TSS and intron 1, suggesting a potential role in locus-specific recruitment of the remodelling complex. Co-occupancy by RNAPII, and association of activating histone modifications, at the *Wt1* ECRs suggests the ECRs are *bona fide* epicardial regulatory elements. By making use of transient transgenic systems and combined reporter-assays, we confirm *in vivo* the transcriptional activity of the *Wt1* ECRs, which is also augmented by Tβ4, further supporting SWI/SNF-mediated function to be significantly enhanced by Tβ4. Chromatin immunoprecipitation-sequencing (ChIP-seq) studies reveal BRG1 occupancy at further epicardial loci, including *Raldh2 (*gene alias *Aldh1a2*), *Tbx18* and *Tcf21*, suggesting a wider role for BRG1–SWI/SNF complexes in the regulation of epicardial activation. Altogether, these findings reveal insight into the function of SWI/SNF in regulating the *Wt1* transcriptional landscape as an important mediator of EPDC activity during development and disease.

## Results

### SWI/SNF catalytic subunits are expressed in the epicardium

**Figure 1:**
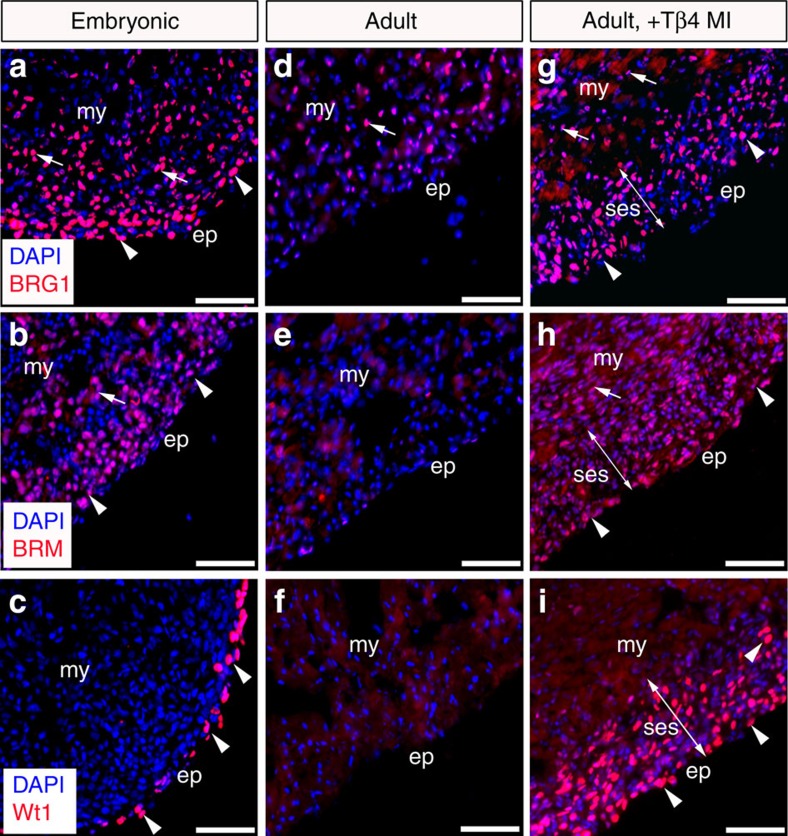
BRG1 and BRM are expressed in the developing and adult epicardium. (**a**–**i**) Immunostaining for BRG1, BRM and Wt1 in mouse E11.5 (**a**–**c**), adult (**d**–**f**) and adult primed- (+Tβ4), -injured (day 4 post-MI) hearts (**g**–**i**). Arrows mark myocardium, arrowheads mark epicardium and double-arrow indicates the expansion of the subepicardial space post-MI. ep, epicardium; my, myocardium; ses, subepicardial space. All scale bars 100 μm.

We found that BRG1 and BRM were localized throughout the developing heart (Fig. 1a,b; Supplementary Fig. 1a–d) and notably were expressed in the epicardium overlapping with the expression domain of Wt1 (Fig. 1c; Supplementary Fig. 1b–e). In the adult heart, BRG1 expression was comparatively reduced in the myocardium and absent from the quiescent epicardium (compare Fig. 1a with Fig. 1d; Supplementary Fig. 1a,b). Moreover, BRM localization was excluded from the epicardium (compare Fig. 1b with Fig. 1e), which also lacked Wt1 expression, consistent with the reported down-regulation of the embryonic epicardial gene program^24^ (Fig. 1f; Supplementary Fig. 1b). However, in response to MI, BRG1 and BRM expression in the epicardium increased (Supplementary Fig. 1f,g), most notably in the expanded subepicardial region (ses; Fig. 1g,h) and co-localized with Wt1 re-expression. Wt1 expression was further enhanced by stimulation with Tβ4 (priming) before MI^24^ (compare Fig. 1i with Supplementary Fig. 1f,h). Taken together, the spatiotemporal expression of BRG1 and BRM and colocalization with Wt1, suggests that SWI/SNF complexes may regulate epicardial *Wt1* expression and, subsequently, EPDC activation. In support of this, EPDCs expressing enhanced GFP from the *Wt1* locus of *Wt1*^*GFPCre/+*^ knock-in mice^17^ were positive for BRG1 and BRM, in both the developing and post-MI adult heart (Supplementary Fig. 1c–h).

### SWI/SNF complex binds regulatory elements in the *Wt1* locus

**Figure 2:**
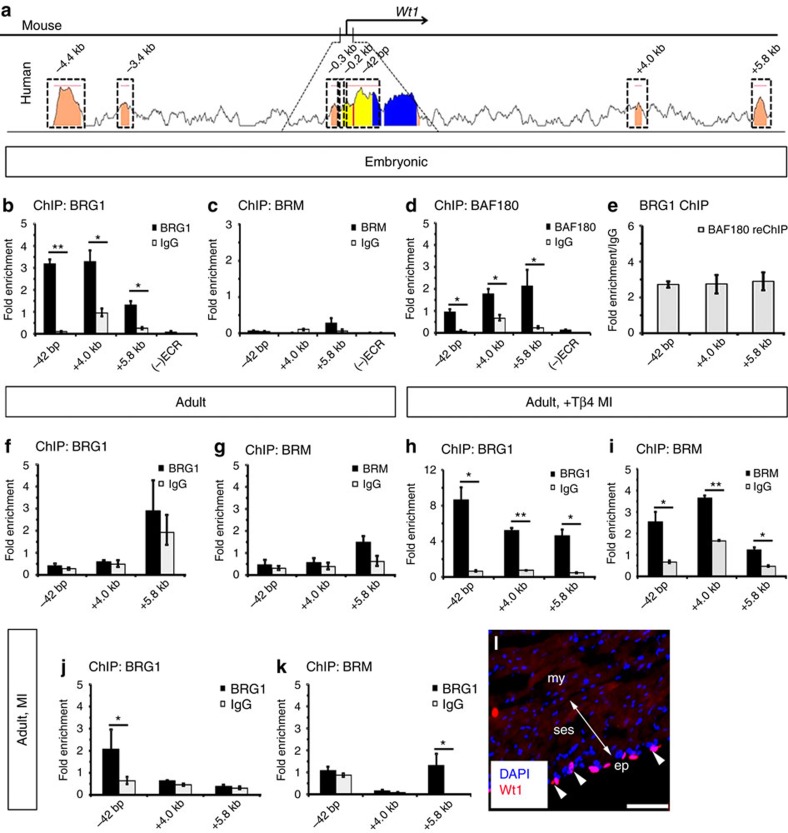
ATP-dependent SWI/SNF complexes bind ECRs in the *Wt1* locus. (**a**) Sequence alignment of the *Wt1* locus from mouse and human. Peak heights indicate degree of sequence homology; pink bars above the peaks denote evolutionary conserved regions (ECRs); yellow represents the 5′ untranslated region (UTR); blue indicates exon 1. Boxed regions were selected for further analysis by ChIP. Numbers above boxed regions denote ECR approximate distances upstream (−) or downstream (+) of the transcription start site (TSS). (**b**–**d**) ChIP-qPCR data using chromatin derived from embryonic hearts at E11.5 and anti-BRG1, anti-BRM or anti-BAF180 antibodies. Note that no enrichment was detected in non-ECR elements ((−)ECR) confirming ChIP signal specificity. (**e**) Sequential ChIP (re-ChIP) with an anti-BAF180 antibody using embryonic heart-derived chromatin pulled-down with anti-BRG1. (**f**,**g**) ChIP-qPCR data using chromatin derived from adult intact (no MI) hearts and anti-BRG1 or anti-BRM antibodies. (**h**,**i**) ChIP-qPCR data using chromatin derived from adult primed- (+Tβ4), injured-hearts at day 4 post-MI and anti-BRG1 or anti-BRM antibodies. (**j**,**k**) ChIP-qPCR data using chromatin derived from adult non-primed, injured-hearts at day 4 post-MI and anti-BRG1 or anti-BRM antibodies. (**l**) Wt1 immunostaining in post-MI adult hearts showing expanded subepicardial space (indicated by double-arrow), but weak re-expression of Wt1 in the epicardium (arrowhead). Three independent experiments per antibody were performed using 20 hearts at E11.5 and three adult hearts per experiment. ChIP results are presented as fold enrichment over input, whereas re-ChIP results are present in fold enrichment over the level of ChIP with negative control IgG antibody. All error bars are data±s.d. Significant differences (*P* values) were calculated using two-tailed Student’s *t*-test (*P≤0.05; **P≤0.01). bp, base pairs; kb, kilobases; my, myocardium; ses, subepicardial space. Scale bar 100 μm.

To determine whether SWI/SNF directly regulates Wt1 expression, we examined BRG1 and BRM binding to the *Wt1* promoter *in vivo*. Using sequence alignment software (www.dcode.org), we compared mouse and human genomes to identify evolutionary conserved regions that may act as sites for SWI/SNF association^7^,^8^ and found seven ECRs in the *Wt1* locus, which were named according to their location in relation to the TSS: two distal, located upstream of the TSS (starting at ≈−4.4 kb and −3.4 kb and extending for 393 bp and 126 bp, respectively), three proximal, located immediately upstream of the TSS (starting at ≈−0.3 kb, −0.2 kb and −42 bp and extending for 101 bp, 129 bp and 439 bp, respectively) and two located within intron 1 (starting at≈+4.0 kb and +5.8 kb and extending for 106 bp and 188 bp, respectively; Fig. 2a; Supplementary Table 1). ChIP on embryonic hearts with an anti-BRG1 antibody revealed that, of the seven ECRs, BRG1 was strongly associated with the proximal promoter (−42 bp) and intronic (+4.0 kb and +5.8 kb) ECRs of *Wt1*, but not with a non-ECR element (-ECR) localized upstream of the TSS (starting at≈−3.8 kb and extending for 100 bp) confirming specificity of the ChIP signal (Fig. 2b). BRM, in contrast, only weakly associated with the +5.8 kb ECR (Fig. 2c), whereas BAF180 revealed a similar binding pattern to BRG1 (Fig. 2d). The co-occupancy patterns observed on *Wt1* ECRs could result from transient binding by separate SWI/SNF components (BRG1 or BAF180) in different subsets of *Wt1*-expressing cells, or could be due to a direct interaction between these proteins. We, therefore, examined potential complex formation at *Wt1* ECRs by sequential ChIP (re-ChIP). Strong re-ChIP signals were detected with an anti-BAF180 antibody on BRG1-enriched *Wt1* ECRs (Fig. 2b) in hearts at embryonic day (E) 11.5 (Fig. 2e), confirming SWI/SNF complex formation. In combination, the ChIP experiments revealed enrichment of SWI/SNF containing BRG1 and BAF180 in regulatory regions of murine *Wt1* in the embryonic heart, which directly correlated with epicardial-specific Wt1 expression (Fig. 1c). Moreover, even though BRM is expressed in the developing epicardium (Fig. 1b), BRG1 is the preferred ATPase subunit of the SWI/SNF complex binding at the *Wt1* locus.

To investigate whether *Wt1* is a direct BRG1–SWI/SNF target in the adult epicardium, we performed ChIP experiments using intact (Fig. 2f,g) and injured-adult hearts (Fig. 2h–l). The adult heart does not ordinarily express Wt1 (Fig. 1f; Supplementary Fig. 1b) and neither BRG1, nor BRM associated with *Wt1* ECRs in the intact adult heart (Fig. 2f,g), as expected given the lack of expression of BRG1 and BRM in adult epicardium (Fig. 1d,e). However, in primed- (+Tβ4) and injured-adult hearts with evident Wt1 re-expression at day 4 post-MI (arrowheads in Fig. 1i), BRG1 had a similar binding pattern to that in the embryonic heart (compare Fig. 2b,h). BRM was also found to bind to −42 bp, +4.0 kb and +5.8 kb ECRs (Fig. 2i), in contrast to the lack of binding in embryonic hearts and suggesting that in the reactivated adult epicardium BRM may act redundantly with BRG1 to regulate *Wt1* expression. By contrast, in non-primed-, injured-adult hearts that exhibited low levels of Wt1 at day 4 post-MI (arrowheads in Fig. 2l) there was minimal association of BRG1 and BRM to *Wt1* ECRs (Fig. 2j,k), even though BRG1 and BRM were expressed (Supplementary Fig. 1b,f,g). Overall, these data demonstrate the existence of ECRs in the *Wt1* locus that are bound by SWI/SNF complexes containing BRG1 or BRM (adult epicardium only) as the catalytic subunit. Occupancy at these ECRs dynamically alters during embryogenesis into adulthood and responds to exogenous Tβ4 and injury, suggesting that chromatin–remodelling of the *Wt1* locus by SWI/SNF is amplified by Tβ4 and directly associates with enhanced *Wt1* and epicardial activity^23^,^24^.

To further characterize the remodelling of the *Wt1* locus in the developing and adult heart, we investigated the association of the −42 bp, +4.0 kb and +5.8 kb ECRs, with RNAPII and canonical epigenetic modifications in histone H3 tails, previously implicated in gene activation and repression: K4me1, K4me3, K27ac and K27me3 (ref. 25). When *Wt1* was ‘off’ (that is, intact adult heart) the repressive H3K27me3, catalysed by the Polycomb complex 2 (PRC2)^26^, was the only epigenetic mark found to significantly associate to −42 bp, +4.0 kb and +5.8 kb ECRs (compare Supplementary Fig. 2e with Supplementary Fig. 2a–d; *P*≤0.05 for the three *Wt1* ECRs; the two-tailed Student’s *t*-test was used to determine the statistical significance). In contrast, when *Wt1* was ‘on’ (that is, embryonic and primed-, injured-adult hearts) RNAPII phosphorylated at serine five (confined to promoter regions and required for the initiation of transcription^27^), was enriched in −42 bp and +4.0 kb ECRs (Supplementary Fig. 2f), suggesting that these elements act as *bona fide* regulatory elements of gene expression. Moreover, H3K4me1 (associated with putative enhancers) was enriched in +4.0 kb and +5.8 kb ECRs (Supplementary Fig. 2g), whereas H3K4me3 (associated with active promoters) was enriched in all three *Wt1* ECRs (Supplementary Fig. 2h). Finally, while the activating histone mark H3K27ac (associated with TSS and active enhancers) was strongly enriched in −42 bp and +4.0 kb ECRs (Supplementary Fig. 2i), the counteracting, mutually exclusive modification H3K27me3 (associated with inactive promoters) was also detected in the three ECRs (Supplementary Fig. 2j), suggesting differences in the epigenetic remodelling of the *Wt1* locus, which likely reflects the dynamic changes in spatiotemporal expression. Of note, when *Wt1* activation is ‘low’ (that is, non-primed, injured-adult hearts) a similar epigenetic profile was observed on *Wt1* ECRs (Supplementary Fig. 2k–o), suggesting that cardiac injury alone, which leads to minimal association of BRG1 and BRM to *Wt1* ECRs (Fig. 2j,k), is sufficient to induce remodelling of the *Wt1* locus and subsequent low level *Wt1* re-expression (Fig. 2l), however, this is suboptimal when compared with ectopic Tβ4 treatment (Fig. 1i).

### C/EBPβ recruits the SWI/SNF complex into the *Wt1* locus

Targeting of the SWI/SNF complex to the *Wt1* locus within the epicardium is likely mediated by sequence-specific transcription factors, which access silent chromatin and initiate cell specific gene activation through recruitment of chromatin–remodelling complexes and co-activator partners^3^. To this end, the pioneering C/EBPs have been shown to mediate epicardial gene activation during heart development and injury^28^. In addition, the C/EBPβ isoform has been shown to recruit the SWI/SNF complex to specific genomic loci, via direct interaction with BRG1 (ref. 29). *In silico* analysis of *Wt1* −42 bp, +4.0 kb and +5.8 kb ECR sequences predicted the existence of C/EBPβ binding sites (Supplementary Table 2), raising the possibility that C/EBPβ may recruit SWI/SNF complexes containing BRG1 to the *Wt1* ECRs. To explore this further, we performed ChIP with an anti-C/EBPβ antibody and observed enrichment of C/EBPβ in all three ECRs in fetal and primed-, injured-adult hearts (Supplementary Fig. 3a–d). Subsequent re-ChIP analyses with an anti-BRG1 antibody confirmed complex formation between C/EBPβ and BRG1 on *Wt1* ECRs (Supplementary Fig. 3b,d). Furthermore, siRNA-targeting of *C/EBPβ* transcripts resulted in decreased expression of *Wt1* and the *Wt1*-downstream target gene *Raldh2* (ref. 21) in mouse primary epicardial cells (Supplementary Fig. 3e,f), supporting a role for a C/EBPβ-SWI/SNF remodelling complex in the regulation of *Wt1* ECRs.

### *Wt1* ECRs drive epicardial gene expression *in vivo*

**Figure 3:**
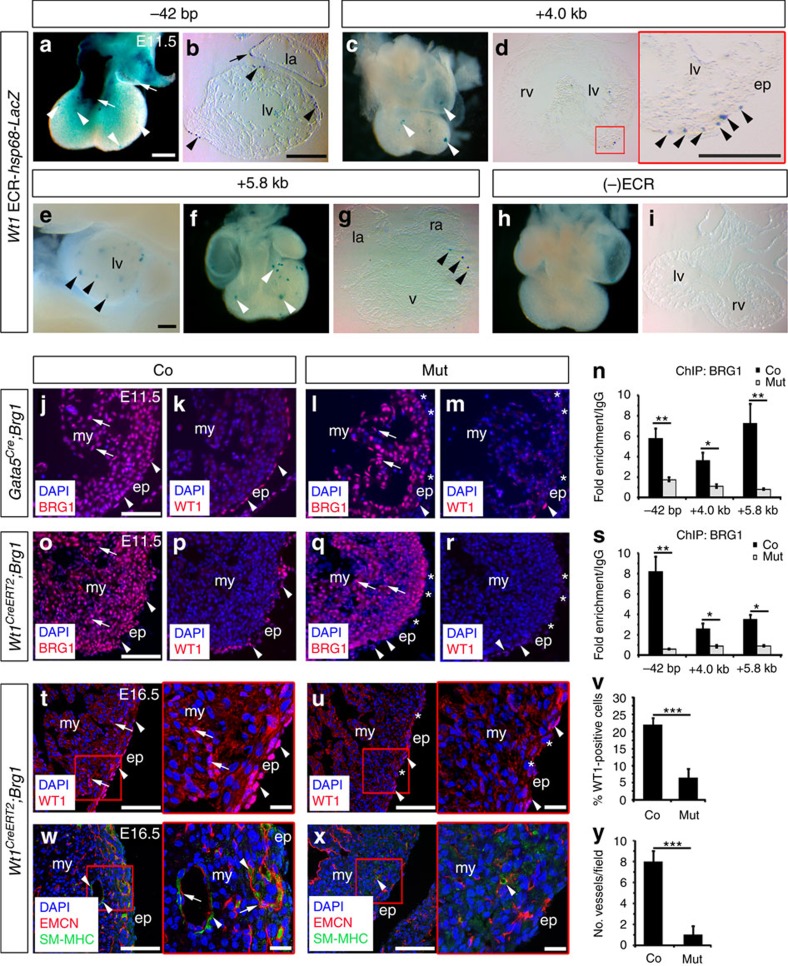
*Wt1* ECRs drive gene expression and BRG1 is required for EPDC activity. (**a**–**i**) *Wt1* ECRs are sufficient to drive *LacZ* expression in the epicardium at E11.5. Whole-mount (**a**,**c**,**e**,**f**,**h**) and transverse-sectioned hearts (**b**,**d**,**g**,**i**) are shown. Arrowheads mark epicardium covering the ventricles; arrows mark epicardium covering the atria (**a**,**b**) and outflow tract (**a**). Two (−42 bp), five (+4.0 kb) and six (+5.8 kb) transgenic mice were examined to assess the reproducibility of reporter activity patterns. (**j**–**s**) BRG1 and Wt1 immunostaining of E11.5 hearts documenting *Brg1* deletion (asterisks in **l**,**q**) and Wt1 reduction (asterisks in **m**,**r**) in the epicardium of *Brg1* cKOs compared to littermate controls (arrowheads in **j**,**k** and **o**,**p**). Note reduced BRG1 expression also in the myocardium (arrows; compared **j** with **l** versus **o** with **q**) of *Gata5*^*Cre*^*;Brg1*^*flox/flox*^, but not *Wt1*^*CreERT2*^*;Brg1*^*flox/flox*^ hearts. (**n**,**s**) BRG1 ChIP-qPCR data. Three independent ChIP experiments were performed per group (*n*=10 hearts) and are presented as fold enrichment over IgG. (**t**,**u**) Wt1 immunostaining of E16.5 hearts documenting fewer Wt1-positive cells in the epicardium (arrowheads) and EPDCs invading the subepicardial and myocardial layers (arrows) in *Brg1* cKOs. Note magnified views of (red) inset boxes in **t** and **u**. (**v**) Quantification of Wt1-expressing cells in E16.5 hearts as %=(total number of Wt1-positive cells per section/total number of DAPI nuclei per section) × 100; co, *n*=3 hearts; mut, *n*=4 hearts. (**w**,**x**) Endomucin (EMCN) and smooth-muscle myosin heavy chain 11 (SM-MHC) immunostaining of E16.5 hearts documenting a reduction of patent coronary vessels, containing endothelium (arrow) surrounded by smooth muscle (arrowheads), in *Brg1* cKOs hearts. Note magnified views of (red) inset boxes in panels **w** and **x**. (**y**) Quantification of patent vessels per field in E16.5 hearts; 6 fields analysed per section per genotype; co, *n*=3 hearts; mut, *n*=4 hearts. All error bars are data±s.d. Significant differences (*P* values) were calculated using two-tailed Student’s *t*-test (*P≤0.05; **P≤0.01; ***P≤0.001). co, control; ep, epicardium; la, left atrium; lv, left ventricle; mut, mutant; my, myocardium ra, right atrium; rv, right ventricle; v, ventricle. All scale bars 200 μm, except **j**,**o**,**t**,**u**,**w**,**x** 100 μm and magnified views of inset boxes in **t**,**u**,**w**,**x** 20 μm.

To obtain *in vivo* evidence of *Wt1* ECR activity, we employed an established transient transgenic enhancer reporter assay^30^ to engineer *Wt1* ECR-*LacZ* mice and observed robust β-galactosidase (β-gal) activity. The reporting of individual *Wt1* ECR activity lacks cooperative interaction with cis-acting elements (enhancers or negative regulatory regions) as would be associated with the intact gene locus and consequently *LacZ*-reporter expression would not be expected to precisely mimic endogenous gene expression and instead overlap in-part with *Wt1* in the developing embryo^31^,^32^ (Fig. 3a–i; Supplementary Figs 4,5). The −42 bp ECR, which based on its genomic location (Fig. 2a) is predicted to act as a *Wt1* core promoter, recapitulated at least some of the regions of *Wt1* expression (Supplementary Fig. 5), and importantly drove β-gal activity strongly throughout the epicardium covering the developing ventricles, atria and outflow tract (arrowheads and arrows, respectively in Fig. 3a,b; Supplementary Figs 4,5). The observed expression in the outflow tract was considerably elevated and regionally expanded relative to the corresponding *Wt1* expression (Fig. 3a, Supplementary Fig. 5), likely due to the absence of cis-acting repressive elements present in the *Wt1*-locus. Elsewhere in the developing embryo, the −42 bp ECR drove β-gal activity strongly in domains of the spinal cord and brain (arrows in Supplementary Fig. 4a and Supplementary Fig. 5), where lateral motor neurons expressing Wt1 are being formed^32^, and the mesothelium of the body wall and gut, which represent further sites of known Wt1 expression^32^ (arrow in Supplementary Fig. 4b; Supplementary Fig. 5). The activity of the more distal, intronic +4.0 kb (Fig. 3c,d; Supplementary Fig. 4d–f) and +5.8 kb (Fig. 3e–g; Supplementary Fig. 4g–i) ECR transgenes was relatively weaker, when compared to the core promoter element (−42 bp ECR), and restricted to epicardial cell clusters. Of note, the +5.8 kb ECR transgene also drove β-gal activity in the urogenital region (Supplementary Fig. 4j-l), where Wt1 plays a pivotal role in development^31^. In the absence of ECR-*LacZ* transgene integration, no β-gal activity was observed confirming signal specificity (Fig. 3h,i).

### BRG1 is required for activation of the *Wt1* locus

We next tested whether BRG1 plays an essential role in *Wt1* regulation. We first used siRNA to specifically knock-down *Brg1* in mouse primary epicardial cells and observed reduction in the expression of *Wt1* and *Raldh2* (Supplementary Fig. 6a,b). Subsequently, we used *Gata5*^*Cre*^ and *Wt1*^*CreERT2*^ *Cre*-drivers crossed with a *Brg1*^*flox/flox*^ mouse line^17^,^33^,^34^,^35^ to conditionally delete *Brg1* in the mouse epicardium by E11.5 and examined *Wt1* expression (Fig. 3j–r; Supplementary Fig. 6c,d). Both *Cre* strains induced reduction of *Brg1* levels in the developing heart. *Gata5*^*Cre*^ was relatively more efficient (Supplementary Fig. 6c), however, this was explained by *Gata5*^*Cre*^ targeting *Cre* activity additionally in non-epicardial cells (Supplementary Fig. 6e,f), such that BRG1 was reduced in the developing endocardium/myocardium and epicardium as compared to *Wt1*^*CreERT2*^ targeting, which was epicardial-specific, albeit with variable efficiency (compare Fig. 3j,l with Fig. 3o,q). Notably, targeting of *Brg1* led to decreased *Wt1* and *Raldh2* expression in mutant hearts, compared to control littermates (Fig. 3k,m,p,r; Supplementary Fig. 6d). Moreover, reduced *Wt1* expression was strongly associated with reduced binding of BRG1 to *Wt1* ECRs in *Brg1*-deficient hearts (Fig. 3n,s), further supporting that BRG1-occupancy is required for *Wt1* activity during murine epicardial development.

To determine the functional consequences of deleting *Brg1* in the developing epicardium, we extended the phenotypic analysis of *Brg1*-deficient embryos to E16.5 and assessed EPDC mobilization and accompanying coronary smooth muscle differentiation^20^ (Fig. 3t–y; Supplementary Fig. 6g–p). No live embryos positive for the *Gata5*^*Cre*^ allele and bearing two copies of the *Brg1* floxed alleles (*Gata5*^*Cre*^; *Brg1*^*F/F*^: Mut) were collected at E16.5; indeed harvesting of embryos at earlier developmental stages revealed that *Brg1* mutants died between E12.5 and E16.5 (Supplementary Fig. 6g). At E12.5, mutant hearts exhibited lower levels of coronary smooth muscle markers compared to littermate controls (Co) (Supplementary Fig. 6h), including *SM22a* (smooth muscle protein 22-alpha) and *SMAα* (smooth muscle actin, alpha). In addition, endothelial/endocardial markers (Supplementary Fig. 6i) such as platelet and endothelial cell adhesion molecule 1 (*PECAM*) and the tyrosine-protein kinase receptor *Tie2*, and myocardial markers (Supplementary Fig. 6j), such as the myosin heavy chain polypeptides α and β (α*MHC* and β*MHC*, respectively), were also reduced in mutants, reflecting *Cre* activity and the requirement for *Brg1* in both epicardial and non-epicardial compartments (Supplementary Fig. 6e). Moreover, histological characterization of transverse sections from mutant hearts revealed under-developed and disorganized chamber walls, with no clear ventricular septation and dilated atria (compare Supplementary Fig. 6k with 6l). The severity of these cardiac malformations is likely to induce embryonic lethality, preventing the examination of patent coronary vasculature at E16.5. Rare surviving mutants, collected at E14.5 (*n*=8 out of 73 embryos) and E15.5 (*n*=1 out of 16 embryos), revealed small, under-developed hearts (Supplementary Fig. 6m,n), a lack of Wt1-positive epicardial cells and a disorganized endomucin (EMCN)-positive endocardium/endothelium (Supplementary Fig. 6o,p), respectively. Consequently, in order to address the functional consequences of epicardial deletion of *Brg1* on EPDC activity at later stages of embryogenesis, we focused our studies on the characterization of mutant embryos generated using the epicardial-specific *Wt1*^*CreERT2*^ *Cre*-driver. *Wt1*-derived Cre activity was induced by tamoxifen delivery to delete *Brg1* from E10 and while mutant embryos collected at E16.5 appeared grossly normal, immunostaining revealed a reduction in the number of Wt1-positive cells located in the epicardium or invading the subepicardial space and adjacent myocardial layer (arrowheads and arrows, respectively in Fig. 3t,u), which was statistically significant when compared to littermate control hearts (Fig. 3v; *P*≤0.001; the two-tailed Student’s *t*-test was used to determine the statistical significance). Additional immunostaining studies using antibodies against the coronary smooth muscle marker SM-MHC (smooth muscle-myosin heavy chain polypeptide 11) and endothelial marker EMCN, showed a dramatic decrease in the number of patent coronary blood vessels (an endothelial layer surrounded by supporting smooth cells) in embryos positive for the *Wt1*^*CreERT2*^ allele and bearing two copies of the *Brg1* floxed alleles (*Wt1*^*CreERT2*^; *Brg1*^*F/F*^: Mut; Fig. 3w–y), further confirming the requirement for *Brg1*-mediated regulation of EPDC activity in the developing heart.

### Tβ4 interacts with BRG1 augmenting *Wt1* activation post-MI

**Figure 4:**
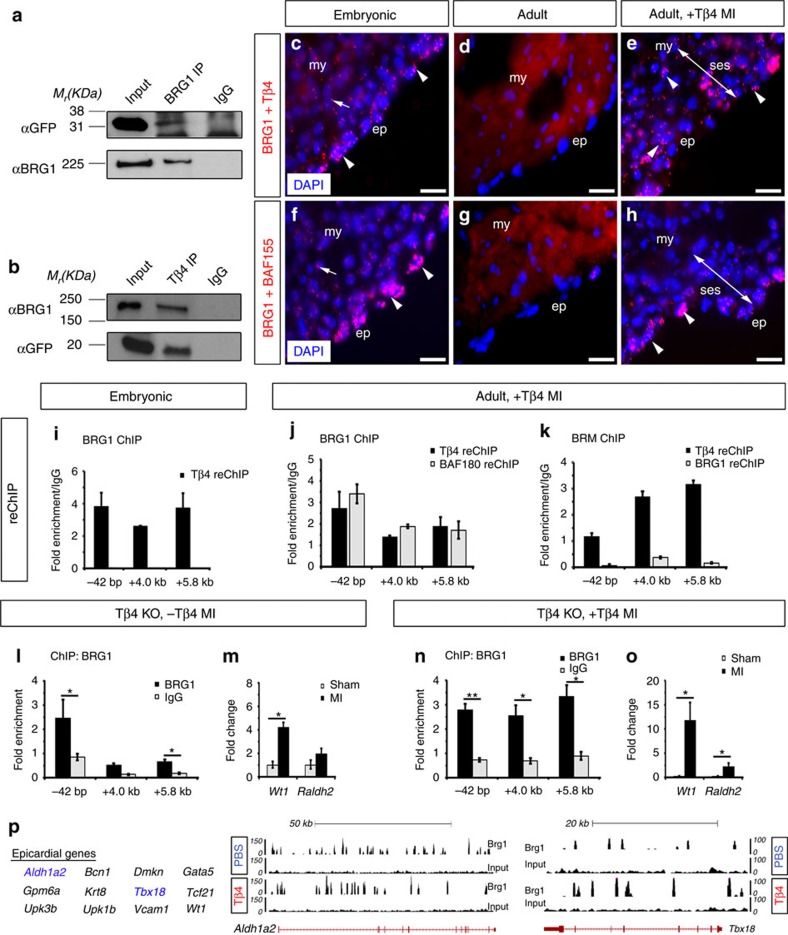
Tβ4 interacts with SWI/SNF enhancing activation of the adult epicardium. (**a**,**b**) Immunoprecipitation experiments with Tβ4-EGFP-transfected cells using anti-BRG1 (**a**) or anti-Tβ4 (**b**) antibodies. The original uncropped images of gels are shown in Supplementary Fig. 11. (**c**–**e**) Duolink *in situ* analysis using anti-rabbit PLUS and anti-mouse MINUS PLA probes specific for Tβ4 and BRG1 antibodies respectively, in embryonic (**c**), intact adult (**d**) and post-MI hearts (**e**). (**f**–**h**) Duolink *in situ* analysis using anti-rabbit PLUS and anti-mouse MINUS PLA probes against BRG1 and BAF155. (**i**–**k**) re-ChIP with anti-Tβ4, anti-BAF180 or anti-BRG1 antibodies using chromatin pulled-down with anti-BRG1 (**i**,**j**) or anti-BRM (**k**) to examine Tβ4-SWI/SNF complex formation in E11.5 (**i**) and adult post-MI, primed hearts (**j**,**k**). (**l**) ChIP-qPCR data from chromatin derived from post-MI, Tβ4 knockout (KO) adult hearts showing minimal binding of BRG1 in *Wt1* ECRs. (**m**) *Wt1* and *Raldh2* expression analysis by qRT-PCR compared to sham-operated animals. (**n**) ChIP-qPCR data from chromatin generated from primed- (+Tβ4), and injured- (MI) adult Tβ4 knockout hearts revealing BRG1 strong enrichment in *Wt1* ECRs. (**o**) *Wt1* and *Raldh2* expression analysis by qRT-PCR compared to sham-operated animals. At least three independent ChIP experiments per antibody per sample group (*n*=10 fetal hearts; *n*=3 adult hearts) were performed. ChIP results are presented as fold enrichment over input, whereas re-ChIP results are present in fold enrichment over the level of ChIP with negative control IgG antibody. (**p**) List of representative BRG1-enriched epicardial genes shared between PBS- and Tβ4-primed, -injured (MI) adult hearts and representative UCSC browser snapshots of the *Aldh1a2* and *Tbx18* loci (highlighted in blue in the list of genes) derived from the Brg1 ChIP-seq experiments. All error bars are data±s.d. Significant differences (*P* values) were calculated using two-tailed Student’s *t*-test (*P≤0.05; **P≤0.01). ep, epicardium; my, myocardium; ses, subepicardial space. All scale bars 50 μm.

Collectively our data revealed that the SWI/SNF complex promotes *Wt1* activity in the developing and adult epicardium, via binding of its catalytic subunit at three discrete *cis*-regulatory elements in the *Wt1* locus. Moreover, SWI/SNF-mediated function is enhanced by stimulation with exogenous Tβ4 before MI. Insight into how Tβ4 amplifies SWI/SNF chromatin–remodelling emerged with the identification of BRG1 as a potential binding partner of Tβ4 via a yeast two-hybrid (Y2H) screen using a mouse embryonic complementary DNA (cDNA) library as prey and Tβ4 as bait to identify molecular interactors. To validate the Y2H finding, we performed co-immunoprecipitation experiments, between EGFP-tagged Tβ4 and endogenous BRG1 (Fig. 4a,b). Tβ4-EGFP co-immunoprecipitated with BRG1, but not with the control IgG indicating specificity of binding (Fig. 4a). Moreover, subsequent immunoblotting for BRG1 confirmed successful immunoprecipitation of the ATPase subunit by the anti-BRG1 antibody (Fig. 4a). Reciprocal co-immunoprecipitation experiments, using an anti-Tβ4 antibody for pull-down with anti-BRG1 and anti-GFP antibodies for immunoblotting, further confirmed physical interaction between BRG1 and Tβ4 (Fig. 4b). Tβ4 is expressed in the developing and adult injured heart, including the epicardium (Supplementary Fig. 1e,h; Supplementary Fig. 7a–c), in an equivalent spatiotemporal pattern to BRG1 supporting a potential protein–protein interaction. To probe a physical association between BRG1 and Tβ4 *in vivo*, we performed Duolink II *in situ* proximity ligation assay (PLA) with specific PLUS and MINUS PLA probes against BRG1 and Tβ4 antibodies in embryonic and postnatal hearts (Fig. 4c–e). Nuclear PLA positive signals (red ‘speckles’) were present in myocardial and epicardial cells in the developing heart (arrow and arrowheads, respectively in Fig. 4c) and in the expanded subepicardial space upon injury (arrowheads in Fig. 4e), but not in the intact adult heart (Fig. 4d). This result was in agreement with the expression pattern of BRG1 (Fig. 1a,d,g). To test the specificity of the nuclear PLA signals both a positive control with BRG1 and BAF155 (another subunit of the SWI/SNF complex, gene alias *SMARCC1*, with a similar expression pattern; Supplementary Fig. 7d–f) antibodies (Fig. 4f–h) and negative controls with Tβ4 and BRG1 antibodies on their own (Supplementary Fig. 7g,h) or BRG1 and Tβ4 antibodies on Tβ4 knockout samples (Supplementary Fig. 7i) were included. Similar to BRG1 and Tβ4, nuclear BRG1 and BAF155 PLA signals were detected exclusively in the myocardium and epicardium of both embryonic and post-MI hearts, confirming the presence of functional SWI/SNF chromatin–remodelling complexes (arrow and arrowheads in Fig. 4f,h). Further confirmation of an interaction between Tβ4 and the SWI/SNF catalytic subunit was obtained *in vivo* by re-ChIP analyses (Fig. 4i,j). Robust re-Chip signals were detected with an anti-Tβ4 antibody on BRG1-enriched *Wt1* ECRs in embryonic (Fig. 4i) and adult primed- (+Tβ4), injured- hearts (Fig. 4j). Of note, the latter BRG1-enriched elements were also enriched for BAF180 (Figs 2e and 4j), confirming the formation of SWI/SNF complexes containing Tβ4, BRG1 and BAF180 in epicardial cells. Likewise, re-ChIP experiments using BRM-enriched *Wt1* ECRs revealed that BRM interacts with Tβ4, but not BRG1 in primed-, injured-adult hearts (Fig. 4k), resulting in the formation of Tβ4–BRM–SWI/SNF complexes in the adult reactivated epicardium. Re-ChIP experiments using BRM-enriched *Wt1* ECRs at E11.5 were not possible due to the low binding of BRM in the *Wt1* locus (Fig. 2c).

Following our observations that Tβ4 priming enhanced SWI/SNF-mediated activation of *Wt1* in the adult epicardium (Fig. 1i versus Fig. 2l; Fig. 2h,i versus Fig. 2j,k; Supplementary Fig. 1b), we tested whether endogenous levels of Tβ4 (Supplementary Fig. 7b,c) were sufficient to promote SWI/SNF complex activity. Tβ4 knockout mice, followed by MI, revealed a modest increase in SWI/SNF-binding *Wt1* ECRs and associated increase in *Wt1* expression during heart injury (Fig. 4l,m; also in development, Supplementary Fig. 8), however, priming with exogenous Tβ4 in Tβ4 knockout mice, followed by MI, resulted in significantly enhanced binding of the SWI/SNF complex to *Wt1* ECRs augmenting *Wt1* reactivation (Fig. 4n,o). This suggested that endogenous Tβ4 is dispensable for optimal SWI/SNF binding and reinforced a functional requirement for ectopic (exogenous) Tβ4 priming in nuclear targeting of SWI/SNF-mediated regulation of *Wt1*. To further investigate the role of Tβ4 in SWI/SNF transcriptional activation of *Wt1*, we cloned the *Wt1* ECRs into a chromatinized episomal reporter, pREP4 (ref. 36), and transfected the constructs into the human cell line SW13 that lacks BRG1 and BRM, but expresses all other SWI/SNF components^8^,^37^. SW13 cells were transfected with luciferase reporters (−0.2 kb-luc, −42 bp-luc, +4.0 kb-luc or +5.8 kb-luc) and either BRG1/BRM/Tβ4 alone or Tβ4 in combination with BRG1 or BRM expression constructs. A myc-tagged Tβ4 construct, carrying a nuclear localization signal (NLS-Tβ4-myc), was used to drive Tβ4 into the nucleus. Restoring BRG1 expression to SW13 cells resulted in a significant increase in activity of all *Wt1* ECR reporters, with the only exception being the −0.2 kb ECR (Supplementary Fig. 9; −0.2 kb-luc: 1.085±0.13 versus −42 bp-luc: 1.211±0.14; +4.0 kb-luc: 1.359±0.11; and +5.8 kb-luc: 1.430±0.20; *P* ≤0.001 (−42 bp-luc), *P≤*0.0001 (+4.0 kb-luc) and *P≤*0.01 (+5.8 kb-luc); one-way analysis of variance (ANOVA) followed up by Tukey’s multiple comparison test was used for comparing three or more groups). A lack of activity at the −0.2 kb ECR was anticipated given that no BRG1 binding was detected for this element, suggesting that it does not convey any regulatory function. Restoring BRM expression also resulted in a significant increase in +4.0 kb-luc and +5.8 kb-luc reporter activity (Supplementary Fig. 9; 1.241±0.07 and 1.376±0.06, respectively, *P* ≤0.01 (+4.0 kb-luc) and *P*≤0.05 (+5.8 kb-luc). Tβ4 expression alone did not induce *Wt1* ECR reporter activity (Supplementary Fig. 9), however, co-expression of BRG1 or BRM with Tβ4 significantly increased −42 bp, +4.0 kb and +5.8 kb-luc reporter activity over BRG1, BRM and Tβ4 alone (Supplementary Fig. 9; −42 bp-luc: 1.406±0.12 (BRG1+Tβ4) and 1.175±0.19 (BRM+Tβ4) versus 1.211±0.14 (BRG1) and 1.055±0.06 (BRM), *P*≤0.001 for both ECR-luc; +4.0 kb-luc: 1.646±0.238 (BRG1+Tβ4) and 1.578±0.11 (BRM+Tβ4) versus 1.359±0.11 (BRG1) and 1.241±0.07 (BRM), *P*≤0.001 and *P*≤0.0001 respectively; +5.8 kb-luc: 1.887±0.414 (BRG1+Tβ4) and 1.684±0.215 (BRM+Tβ4) versus 1.430±0.20 (BRG1) and 1.376±0.06 (BRM), *P*≤0.01 and *P≤*0.05 respectively; one-way ANOVA followed up by Tukey’s multiple comparison test was used for comparing three or more groups). These results are in line with our *in vivo* observations (Fig. 4l–o) and support direct activation of *Wt1* ECRs by SWI/SNF complexes containing Tβ4.

### A role for BRG1-SWI/SNF in global gene activation

To investigate whether the role for BRG1–SWI/SNF complexes is restricted to the *Wt1* locus, or whether they might regulate other epicardial loci, we employed ChIP-seq to perform a comparative analysis of the binding of BRG1 in PBS- versus Tβ4-primed, injured- (MI) adult hearts (Fig. 4p; Supplementary Fig. 10). Among the shared genes, between PBS- and Tβ4-primed, injured-adult hearts, we identified binding at *Wt1*, confirming our targeted ChIP-qPCR data, and at other fetal epicardial loci such as *Tbx18*, *Aldh1a2* and *Tcf21* (Fig. 4p). BRG1 binding was also evident at adult epicardial genes (Fig. 4p) previously identified in a transcriptomic study of the adult murine epicardium^38^, including the membrane proteins uroplakin 3b (*Upk3b*), uroplakin 1b (*Upk1b*), glycoprotein m6a (*Gpm6a*) and the transcription factor basonuclin-1 (*Bcn1*). In addition, key myocardial genes *Myh6* (also known as *αMHC*) and *Myh7* (also known as *βMHC*), encoding for myosin heavy chains alpha and beta, respectively, and other cardiac-related genes, such as *Col1a2* (collagen 1 alpha-2, a fibroblast marker) and *Acta2* (also known as *SM* actin, alpha 2, a smooth muscle marker), were also represented in the list of shared BRG1-enriched genomic regions (Supplementary Fig. 10), suggesting novel gene-regulating functions for the SWI/SNF chromatin–remodelling complex in the (adult) injured heart. Of significance, the expression of some of these genes (for example, *αMHC* and *βMHC*) was found to be decreased upon *Gata5*^*Cre*^-mediated deletion of *Brg1* in the developing non-epicardial compartment (Supplementary Fig. 6h,j), in agreement with the hypothesis that the function of the SWI/SNF chromatin–remodelling complex in the developing heart is recapitulated in the adult injured heart^7^. To validate the genomic binding of BRG1, we analysed the ChIP-seq profile in already described sites of SWI/SNF occupancy, such as our intronic +4.0 kb ECR within the *Wt1* locus (Fig. 2) and the 5 kb region upstream of the *Myh7* TSS^7^. While Brg1-binding was detected in these regions in both PBS- and Tβ4-primed, -injured samples, considerably more peaks were observed in the Tβ4-primed sample set (Supplementary Fig. 10). Likewise, BRG1 peak density was comparatively higher in epicardial loci such as *Tcf21*, *Upk3b* and *Tbx18* and cardiac-related loci such as *Acta2* in the Tβ4-primed, -injured sample set versus the non-primed (PBS) group (Fig. 4p; Supplementary Fig. 10). As such, we speculate that Tβ4 may play a role in amplifying BRG1-targeting of downstream embryonic epicardial loci, as important for inducing the reactivation of EPDCs following adult heart injury. Taken together, our ChIP-seq data supports BRG1–SWI/SNF binding of *Wt1* and suggests a role for chromatin–remodelling in activation of further key epicardial genes, such as *Tbx18* and *Tcf21*.

## Discussion

Here we reveal that epicardial activity is epigenetically regulated during development and adult heart injury and that *Wt1*, the master regulator of epicardial EMT, is dynamically controlled by SWI/SNF chromatin–remodelling complexes containing BRG1 and Tβ4 (Figs 1, 2, 3, 4). Tβ4 lacks a DNA-binding domain, but physically interacts with SWI/SNF (Fig. 4) and serves to augment BRG1 and BAF/BRG1 binding at specific enhancer sites within the *Wt1* locus (Figs 2 and 4). BRG1, as the preferred SWI/SNF ATPase subunit, was essential to maintain *Wt1* expression and associated EPDC activity (Fig. 3). Tβ4–SWI/SNF contained the epicardial-specific pioneer transcription factor C/EBPβ (Supplementary Fig. 3), which likely functions to target the complex to the *Wt1* locus. Tβ4-BRG1 binding may be mediated via nuclear actin as an essential component of the SWI/SNF complex which, along with the BAF53 subunit, tightly binds BRG1 to regulate its ATPase activity^39^. Given the high affinity of Tβ4 for monomeric G-actin^40^ and previous reports of co-localization of Tβ4 with actin in the nucleus^41^,^42^ it is possible that Tβ4 interacts with BRG1 through provision of essential G-actin for complex formation and chromatin remodelling activity; although an actin-independent role for Tβ4 cannot be excluded at this stage.

The reactivation of embryonic genes is a hallmark of attempts by the adult heart to compensate for cardiovascular stress and disease. This includes pathologic hypertrophy which is associated with the up-regulation of a signature fetal gene program^43^. BRG1 is reactivated in the adult myocardium in response to cardiac stress and forms a complex with its embryonic partners, HDACs (histone deacetylases) and PARP (poly ADP ribose polymerase), to induce α-myosin heavy chain (MHC) to β-MHC switch^7^. Fetal gene re-expression, in this setting, is insufficient to counteract the pathology and suppress adverse remodelling and is unable to instigate a regenerative response. There is a requirement, therefore, for amplification of the epigenetic effector signals and to elicit targeting to key loci, upstream of multiple developmental pathways. In this regard, the relative levels of Tβ4 appeared to be critical for efficient SWI/SNF complex activity and *Wt1* expression (Figs 1, 2 and ), which likely sits upstream of EPDC activation and cell fate. This was particularly relevant in the context of reactivation of the adult epicardium upon myocardial injury (Figs 2 and 4), whereby ectopic Tβ4 priming resulted in optimal activation of SWI/SNF-enhanced reactivation of EPDCs (Figs 2 and 4) and subsequent differentiation into coronaries and muscle^23^,^24^; while injury alone (despite inducing an up-regulation of endogenous Tβ4 expression) resulted in minimal SWI/SNF activation (Fig. 2) of EPDCs^24^,^44^, which subsequently adopts a default fibroblast differentiation program^22^. ChIP-seq revealed an extensive range for Tβ4–BRG1–SWI/SNF activity following adult cardiac injury at additional epicardial and cardiac gene loci (Fig. 4). Further studies will be required to dissect the functional implications of promiscuous BRG1–Tβ4 activity in the context of promoting adult cell activation, in light of our previous findings that Tβ4 treatment prior to MI facilitates improved heart repair^2^. Epigenetic induction, with co-factor amplification, of fetal genes to activate adult progenitor populations may represent a general paradigm in cell-based tissue repair. More specifically, targeting dynamic chromatin signatures at key embryonic genes that underlie epicardial cell activation could represent a therapeutic strategy to regenerate the injured-adult mammalian heart.

## Methods

### Animal models

All animal experiments were carried out according to UK Home Office project Licence PPL30/2987 compliant with the UK animals (Scientific Procedures) Act 1986 and approved by the local Biological Services Ethical Review Process. Mice were housed and maintained in a controlled environment. *Gata5*^*Cre*^, *Tβ4*^*−/−*^, *Wt1*^*GFPCre*^, *Wt1*^*CreERT2*^, *Brg1*^*flox/flox*^ and *R26R*^*tdTomato*^ mice were kept in a C57Bl/6J inbred background^17^,^33^,^34^,^35^,^45^,^46^. For timed mating experiments, 8–12 week old mice were set-up overnight and females checked for vaginal plugs the following morning; the date of a vaginal plug was set as embryonic day (E) 0.5. For tamoxifen-dependent tissue-specific gene inactivation using the *Wt1*^*CreERT2*^ driver, 2 mg of 4-hydroxytamoxifen (Sigma) were administered to pregnant mice by intraperitoneal (i.p.) injection at both E9.5 and E10.5 to maximize Cre-ERT2 activity in the developing epicardium.

### Thymosin β4 administration

The injection regimen of Tβ4 for priming of wild-type C57BL/6J, *Tβ4*^*−/−*^ and *Wt1*^*GFPCre/+*^ mice have been previously described^24^. Briefly, adult mice received i.p. injection of clinical grade Tβ4 (12 mg/kg; RegeneRex Biopharmaceuticals Inc.) or vehicle (PBS) daily for 7 days. On the eighth day, MI was performed and further injections of Tβ4/vehicle were given on day 0 and day 2 post surgery.

### Myocardial infarction

All surgical and pharmacological procedures were performed in accordance with the Animals (Scientific Procedures) Act 1986 (Home Office, UK). For MI experiments, adult mice (12–14 week old; 25–30 g body weight) were primed with Tβ4 or vehicle, as described above, before surgical procedures. MI was induced in isoflurane-anaesthetized mice by permanent ligation of the left anterior descending coronary artery (LAD). For sham controls, a suture was passed under the LAD but not ligated. Upon recovery (day 0), animals received i.p. injection of Tβ4 (12 mg kg^−1^) or vehicle (PBS). Further injections were given every second day. Hearts were harvested at 4 days following ligation.

### Immunodetection methods

Cryosections were processed for indirect immunofluorescence using standard methods. AlexaFluor secondary antibodies (Invitrogen, 1:200) were used in all cases. Where mouse primary antibodies were used, 1 h incubation with AffiniPure Fab Fragment Goat Anti-Mouse IgG (Jackson ImmunoResearch, 1:50) before primary antibody incubation was performed to reduce non-specific signal. Imaging was performed using a Zeiss Axio Imager Z1 microscope with an AxioCam MRm camera attachment running AxioVision software release 4.8 (Carl Zeiss).

### Duolink *in situ* analysis

Cryosections were processed as for immunofluorescence, up to and including Fab fragment treatment and incubation with primary antibodies. Duolink was performed as per manufacturer’s instructions (Olink Bioscience) using anti-rabbit PLUS and anti-mouse MINUS PLA probes and Orange detection reagents. Imaging was performed using a Zeiss Axio Imager Z1 microscope with an AxioCam MRm camera attachment running AxioVision software release 4.8.

### Whole-embryo *in situ* hybridization

RNA *in situ* hybridization on whole-embryo was performed on E11.5 embryos fixed with 4% formaldehyde solution. In brief, embryos were treated with proteinase K for 30 min and post-fixed with 4% formaldehyde solution and 0.2% glutaraldehyde. The embryos were then incubated with digoxigenin (DIG)-labelled riboprobes transcribed from cDNA-containing plasmids at 65 °C overnight. In the next day, the embryos went through stringent washes in decreasing concentrations of saline-sodium citrate buffer. The DIG probe was then detected with anti-DIG-alkaline phosphatase (AP) Fab fragment (Sigma-Aldrich) by incubating at 4 °C overnight. Colour was developed by incubating embryos in BM Purple (AP substrate; Sigma-Aldrich) at pH9.5 until the pattern was clear. The plasmid encoding *Wt1* was kindly provided by Mathilda Mommersteeg (Oxford, UK).

### Cell culture

HEK293 cells (ATCC CRL-1573) were cultured in DMEM/F12+Glutamax supplemented with 10% FCS and 1% penicillin-streptomycin (Life Technologies); SW13 cells (ATCC CLL-105) were cultured in Leibovitz L-15 (Life Technologies) supplemented with 10% FBS and penicillin-streptomycin; mouse primary epicardial cells were culture in DMEM+Glutamax supplemented with 10% heat-inactivated FCS, 1% streptomycin (Life Technologies), insulin-transferrin-selenium (Biosource) and 10 units ml^−1^ mouse gamma interferon (PeproTech). Cells were maintained in a humidified atmosphere of 5% CO_2_ at 37 °C (HEK293 and SW13 cells) or 33 °C (mouse primary epicardial cells) and were passaged at 80–90% confluence using 0.05% trypsin (Life Technologies). All cell lines used in this study have been routinely tested and found to be negative for mycoplasma contamination.

### Protein extraction

To prepare protein samples from cells for co-immunoprecipitation and/or sodium dodecyl sulfate polyacrylamide gel electrophoresis (SDS–PAGE), cells were lysed in 150 mM NaCl; 20 mM Tris–HCl pH 7.5; 1% Triton X-100; 2 mM EDTA; 1 × protein inhibitor cocktail (Roche). Lysates were incubated on ice for 20 min, centrifuged at 16,000*g* at 4 °C, and supernatants prepared for the relevant application. To prepare protein samples from adult hearts for SDS–PAGE, hearts were collected and snap frozen before being manually powdered in liquid nitrogen using a pestle and mortar. The powdered tissue was transferred into a pre-cooled 1.5 ml tube and lysed in 1 ml of RIPA buffer (50 mM Tris–HCl pH 7.6; 150 mM NaCl; 1% NP-40 (Calbiochem); 0.5% sodium deoxycholate; 0.1% SDS; 1 × Protease Inhibitor Cocktail (Roche); 1 mM PMSF (Sigma); 1 μg ml^−1^ aprotinin (Sigma)) using a 21G needle and 1 ml syringe. Samples were left to incubate on ice for 30 min, with vortexing every 10 min, and then centrifuged at 16,000*g* for 20 min. Supernatants were then prepared for SDS–PAGE by the addition of 2 × Laemmli sample buffer (125 mM Tris pH 6.8; 4% SDS; 25% glycerol; 0.1% bromophenol blue; 5% β-mercaptoethanol) and boiling at 95 °C for 5 min.

### Co-Immunoprecipitation and immunoblotting

For the co-IP experiments (Fig. 4a,b), HEK293 cells were transfected using Polyfect Transfection Reagent (Qiagen). Approximately 2.4 × 10^6^ cells were seeded into 100 mm tissue culture dishes and transfected 24 h later with 8 μg of Tβ4–EGFP–N3 and 80 μl Polyfect. After 48 h of post-transfection, cells were lysed as detailed above and 750 μl of lysate was used for immunoprecipitation (IP) using Dynabeads Protein G (Invitrogen), as per manufacturer’s instructions but with amendments to the incubation times. Antibodies used for IP were: Brg1-H88 (Santa Cruz); Tβ4 (Immundiagnostik); control Rabbit IgG (Abcam). Briefly, 3 μg antibody diluted in 200 μl PBS+0.02% Tween-20 were added to 50 μl Dynabeads and incubated with rotation for 2 h at room temperature. Beads were separated using a DynaMag-2 magnet (Invitrogen) and washed once with PBS+0.02% Tween-20. Lysates were added to the antibody-bound beads and incubated with rotation overnight at 4 °C. The beads were washed 4 times with PBS and suspended in 20 μl 2 × Laemmli sample buffer (125 mM Tris pH 6.8; 4% SDS; 25% glycerol; 0.1% bromophenol blue; 5% β-mercaptoethanol) boiled at 95 °C for 5 min and the supernatant collected and analysed by SDS–PAGE. Lysate not used for IP (‘INPUT’) was processed for SDS-PAGE by the addition of 2 × Laemmli sample buffer and boiling at 95 °C for 5 min. The original uncropped images of gels are shown in Supplementary Fig. 11.

Western blotting (Supplementary Fig. 1b) was performed using standard SDS–PAGE methods using HRP-conjugated secondary antibodies and enhanced chemiluminescence detection (both GE Heathcare). The original uncropped images of gels are shown in Supplementary Fig. 12.

### Dual luciferase assays

Transfection of SW13 cells for luciferase assays was performed using Lipofectamine-2000 (Invitrogen) as per manufacturer’s instructions. Briefly, 1 × 10^5^ cells were seeded into 24 well plates in antibiotics-free medium, and transfected 24 h later with 0.8 μg DNA, comprising 0.45 μg firefly luciferase in pREP4, 0.05 μg TK-Renilla luciferase in pREP7 and 0.3 μg test DNA (when transfecting 2 test constructs, a 1:1 ratio was used; when using only 1 test construct, the remaining DNA was made up with empty pCI vector, which also acted as ‘control’ DNA) and 2 μl Lipofectamine-2000. Cells were lysed 48 h post-transfection and processed for the Dual-Luciferase Reporter Assay System (Promega), as per manufacturer’s instructions. The ratio of Firefly:Renilla luminescence was used to compare samples. All transfections and subsequent readings were performed in triplicate.

### DNA constructs and plasmids

Sanger sequencing (Source Bioscience) was performed on all DNA constructs. Tβ4-EGFP-N3: using a full-length cDNA of Tβ4 in pcDNA3.1 as template DNA, PCR was performed using primers that added a Kozak sequence and *EcoRI* site to the 5′ end and a flexible glycine-serine linker and *BamHI* site to the 3′ end of the Tβ4 open-reading frame (ORF). PCR was carried out using *Pfu* polymerase (Promega). The PCR product was digested with *EcoRI* and *BamHI* and ligated into pEGFP-N3 using T4 ligase (Promega). For NLS-Tβ4-myc/pcDNA3: NLS-Tβ4-myc was synthesized by Eurofins MWG Operon and inserted into pcDNA3. For −0.2 kb-luc/pREP4: pREP4-luc was provided by Keji Zhao (NIH, Bethesda). Wt1^767^-luc/pGL2 Basic – a 767 bp sequence of the mouse *Wt1* promoter spanning −513 to +254 nucleotides relative to the transcription initiation site was provided by Holger Scholz (Berlin). Using Wt1^767^-luc/pGL2 Basic as template, ECR −0.2 kb was amplified using PCR with primers that added a *BglII* site to the 5′ end and an *XhoI* site to the 3′ end (NB. same primers as used for ChIP-qPCR (Supplementary Table 3), but relevant restriction sites were inserted in the forward and reverse sequences to allow cloning of ECR). PCR was carried out using *Pfu* polymerase. The PCR amplicon was purified, digested and was ligated to pREP4-luc. For −42 bp-luc/pREP4: using Wt1^767^-luc/pGL2 Basic as template, ECR −42 bp was amplified using PCR with primers that added a *BglII* site to the 5′ end and an *XhoI* site to the 3′ end (NB. same primers as used for ChIP-qPCR (Supplementary Table 3), but relevant restriction sites were inserted in the forward and reverse sequences to allow cloning of ECR). PCR was carried out using *Pfu* polymerase. The PCR amplicon was purified and digested with *BglII* and *XhoI* and was ligated to pREP4-luc. For +4.0 kb-luc/pREP4: using *Wt1* intron 1/pGL3 Basic as template (Christoph Englert, Jena), the intrinsic ECR +4.0 kb was amplified using PCR primers that added a *KpnI* site to the 5′ end and an *XbaI* site to the 3′ end (NB. same primers as used for ChIP-qPCR (Supplementary Table 3), but relevant restriction sites were inserted in the forward and reverse sequences to allow cloning of ECR). PCR was carried out using *Pfu* polymerase. The SV40 promoter from pGL3-promoter was excised by sequential *XhoI* and *HindIII* digestion, with the *XhoI* site blunted using T4 polymerase (Invitrogen), and ligated into the promoterless pREP4-luc, cut sequentially with *NotI* and *HindIII*, with the *NotI* site blunted using T4 polymerase. ECR +4.0 kb was digested with *KpnI* and *XbaI* and ligated into SV40 promoter-pREP4-luc cut with *KpnI* and *NheI*, thereby placing ECR +4.0 kb upstream of the SV40 promoter. For +5.8 kb-luc/pREP4: using *Wt1* intron 1/pGL3 Basic as template, the intrinsic ECR +5.8 kb was amplified using PCR primers that added a *NheI* site to the 5′ end and a *BglII* site to the 3′ end (NB. same primers as used for ChIP-qPCR (Supplementary Table 3), but relevant restriction sites were inserted in the forward and reverse sequences to allow cloning of ECR). PCR was carried out using *Pfu* polymerase. The PCR amplicon was digested with *BglII* and *NheI* and ligated into pGL3-Promoter, also cut with *BglII* and *NheI*, thereby placing ECR +5.8 kb upstream of the SV40 promoter. ECR +5.8 kb—together with the vector’s SV40 promoter and luciferase—was then excised from pGL3-promoter using *NheI* and *SalI* and ligated into pREP4 (now depleted of its luciferase). For TKRenilla/pREP7: the Renilla luciferase under control of the TK promoter was cut from pRL-TK (Promega) by sequential *BglII* and *BamHI* digestion, with the *BglII* site blunted using T4 polymerase and ligated into pREP7 (Invitrogen) cut sequentially with *XbaI* and *BamHI*, with the *XbaI* site blunted using T4 polymerase, thereby removing pREP7’s RSV promoter. Brg1/pCI: human Brg1 in pCI was a kind gift from Anthony Imbalzano (Worcester, MA). Brm/pCI: human Brm in pCI was a kind gift from Samisubbu Naidu (Indianapolis, IN).

### Generation of *Wt1* ECR*-lacZ* transgenic mice

*In vivo* validation of *Wt1* −42 bp, +4.0 Kb and +5.8 Kb ECRs activity was done using an established mouse transgenic system^30^, where a vector containing the cloned ECR (see DNA constructs and Plasmids), a *Hsp68* basal promoter and a β-galactosidase gene (*LacZ*) is integrated into the mouse genome via standard pronuclear injection of oocytes. Embryos were collected at E11.5 and stained for β-galactosidase (β-gal) activity (see below). Integration of *LacZ* transgenes was further confirmed by PCR analysis.

### β-Galactosidase staining

For β-galactosidase (β-gal) staining embryos were collected at E11.5, fixed on ice for 30 min in 2% formaldehyde solution containing 0.2% glutaraldehyde (both Sigma), washed twice with phosphate-buffered solution (PBS) on ice, and stained overnight at room temperature in X-gal staining solution containing 4 mM K_4_Fe(CN)_6_, 4 mM K_3_Fe(CN)_6_, 2 mM MgCl_2_ and 1 mg ml^−1^ X-gal (dissolved in N-dimethylformamide; Sigma).

### RNA isolation and gene expression profile by qRT-PCR

For postnatal heart samples, total RNA was isolated using Trizol reagent (Invitrogen), according to the manufacturer’s instructions. For fetal hearts or cells, total RNA was isolated using RNeasy Micro Kit (Qiagen), according to the manufacturer’s instructions. Total RNA was reverse-transcribed using oligo dT primers and Superscript III RT (Invitrogen). Real-time RT-PCR analysis was performed on an ABI 7900 Sequence Detector (Applied Biosystems) using SYBR Green (QuantitectTM SYBR Green PCR Kit, Qiagen). Data were normalized to *Hprt* or *18S* (RNA stability assay) expression. Fold-change in gene expression was determined by the 2^−ΔΔCT^ method and is presented relative to levels in sham, E11.5, E12.5 or control siRNA samples. Primer sequences are listed in Supplementary Table 3. Statistical differences were detected using a two-tailed Student’s *t*-test.

### Chromatin immunoprecipitation

Chromatin was prepared from fetal and postnatal hearts (intact and post-MI). Hearts were dissected in chilled PBS; minced and fixed with 1% formaldehyde solution at room temperature, and quenched with 0.125 M glycine before washing twice with PBS supplemented with protease inhibitors (Protease Inhibitor Cocktail Tablet (Roche), 1 mM PMSF (Sigma) and 1 μg ml^−1^ aprotinin (Sigma)). Cells were then lysed (10 mM EDTA, 50 mM Tris–HCl (pH 8.0), 1% SDS, protease inhibitors). Chromatin was sonicated to generate fragment sizes of 500–1000, bp, and immunoprecipitated using antibodies listed in Supplementary Table 4. Isolation of immunoprecipitated chromatin was done according to manufacturer’s protocol (Millipore). The amount of specific immunoprecipitated DNA was then quantified by qPCR (see above) using primers specific for each individual ECR sequence (and, as such, able to distinguish between the different DNA sequences enriched by antibody pull-down), with each reaction performed in triplicate (primers sequence used in ChIP-qPCR are included in Supplementary Table 3). Signals from individual ChIP reactions were used to calculate the % recovery of a given DNA segment relative to the total input signal. Fold enrichment was determined as the fold-change in per cent of input (ChIP signal/input signal) at the target regions (*Wt1* ECRs)^47^. For the data presented in Fig. 3 however, fold enrichment values are presented as the fold-change over the level of ChIP with negative control IgG antibody (ChIP signal/IgG signal) to allow comparison between control (co) and mutant (mut) samples.

### ChIP-sequencing

Brg1 ChIP-seq was performed on PBS- and Tβ4-primed, injured-adult hearts (biological duplicates) collected four days after MI. Briefly, tissues were cross-linked with 1% formaldehyde at room temperature for 10 min, incubated in nuclei extraction buffer first, then in lysis buffer and sonicated. Solubilized chromatin was incubated overnight at 4 °C with a specific rabbit polyclonal anti-Brg1 antibody (Millipore; Supplementary Table 4) coupled to anti-rabbit IgG Dynabeads (Invitrogen). Immunoprecipitated chromatin was then purified by standard phenol-chlorophorm extraction and DNA libraries were prepared with the NEBNext Ultra DNA Library Prep Kit for Illumina according to the manufacturer’s protocol. Libraries were quantified with Qubit dsDNA HS Assay Kit (Invitrogen) and KAPA Library Quantification Kit for Illumina, and then sequenced on an Illumina NextSeq 500 instrument. Sequence reads were mapped with Bowtie (version 1.0.0), using-m 2-X 2000 parameter, to the mouse reference genome (mm9), obtained from the UCSC genome browser. Peaks were called using MACS2 (version 2.0.10) using bdgcomp function. Genes closest to peaks were determined using Bedtools (version 2.17.0). Bigwig files were generated using an enhanced perl script (courtesy of Dr Jim Hughes, Weatherhall Institute of Molecular Medicine, University of Oxford, UK). Plots were made using ChiPSeeker R/Bioconductor package^48^.

### Sequential ChIP

After primary ChIP, specific immunoprecipitates were washed, eluted, incubated with 10 mM dithiothreitol at 37 °C for 30 min, diluted 10 times in ChIP buffer (10 mM EDTA, 50 mM Tris–HCl (pH 8.0), 1% SDS, protease inhibitors) and re-immunoprecipitated with the secondary antibodies. Isolation of re-immunoprecipitated chromatin was done according to manufacturer’s protocol (Millipore). Extracted DNA was used for qPCR (see above). Fold enrichment was determined as the fold-change in per cent IgG (ChIP signal/IgG signal) at the target regions^47^,^49^.

### siRNA experiments

siRNA sequences targeting *Brg1* and *C/EBPβ* transcripts (Silencer Select Pre-designed siRNA from Ambion) were transfected into mouse primary epicardial cells using Lipofectamine 2000 (Life Technologies) according to the manufacturer's instructions.

### Statistical analysis

Animal numbers and sample sizes reflected the minimal number needed for statistical significance based on previous experience. For MI studies, hearts showing no morphological signs of infarct (for example, tissue blanching around the ligating suture) at the stage of sample collection were excluded from further analysis. No randomization or blinding was used. Statistics were calculated using Microsoft Excel and Prism Graphpad software. The statistical significance between two groups was determined using two-tailed Student’s *t*-test (Microsoft Excel), and the data were reported as mean±s.d. Among three or more groups (for example, data shown in Supplementary Fig. 9), one-way analysis of variance (ANOVA) followed up by Tukey’s multiple comparison test was used for comparisons (Prism Graphpad software). A value of *P*<0.05 was considered statistical significant.

### Data availability

The ChIP-seq data that support the findings of this study have been deposited in Gene Expression Omnibus (GEO) with the accession number GSE76239. All other relevant data are available from the corresponding authors on reasonable request.

## Additional information

**How to cite this article:** Vieira, J. M. *et al*. BRG1-SWI/SNF-dependent regulation of the *Wt1* transcriptional landscape mediates epicardial activity during heart development and disease. *Nat. Commun.* **8**, 16034 doi: 10.1038/ncomms16034 (2017).

**Publisher’s note:** Springer Nature remains neutral with regard to jurisdictional claims in published maps and institutional affiliations.

## Supplementary Material

Supplementary Information
