# Supplementary material for: BRG1-SWI/SNF-dependent regulation of the Wt1 transcriptional landscape mediates epicardial activity during heart development and disease
Source: Nat Commun. 2017 Jul 24;8:16034. doi: 10.1038/ncomms16034 (PMC5527284; doi:10.1038/ncomms16034)
Supplement: Supplementary Information [file ncomms16034-s1.pdf]

File Name: Supplementary Information
Description: Supplementary Figures, Supplementary Tables and Supplementary References.

Vieira et al_Supplementary Figure 1

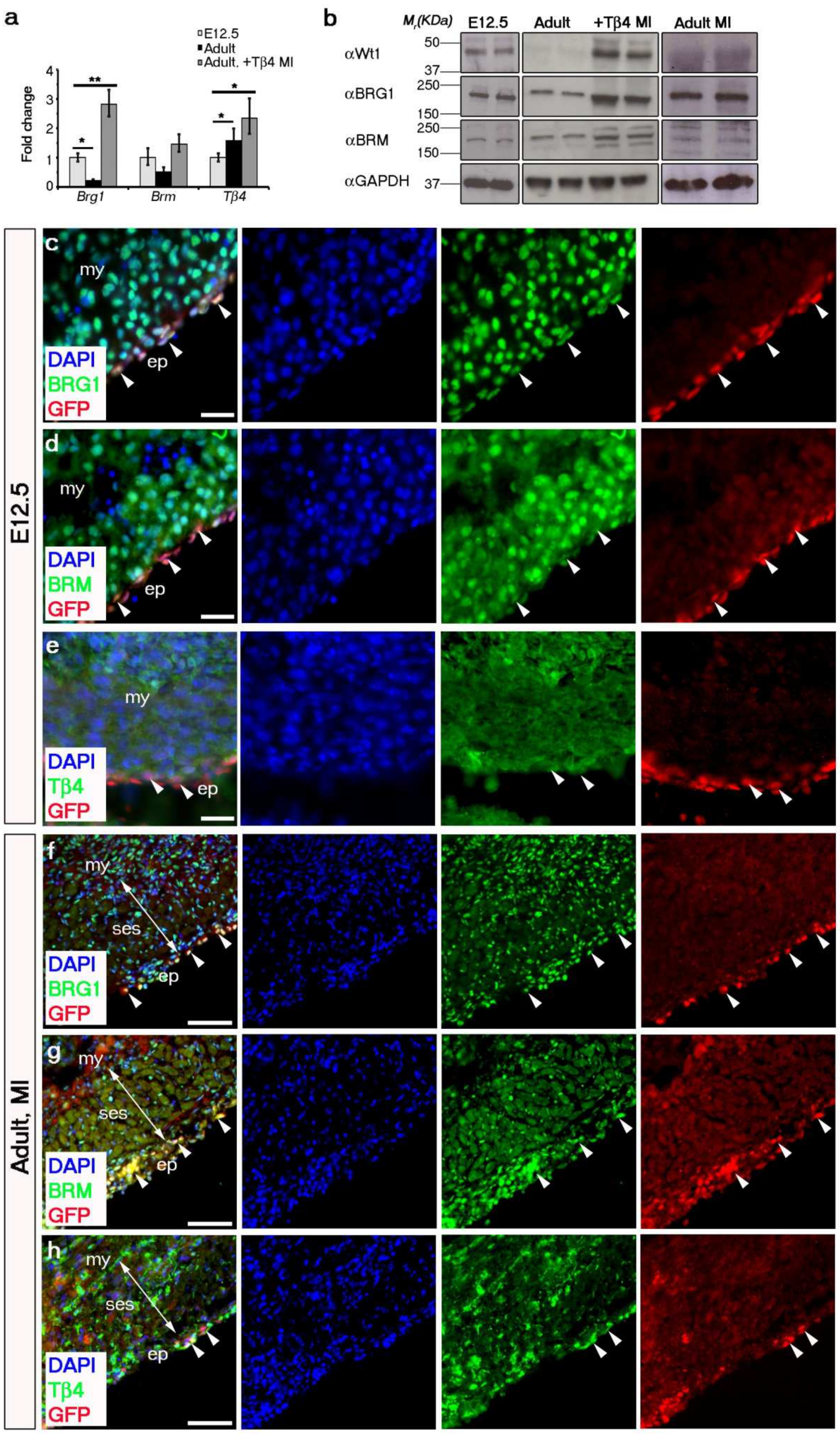

Supplementary Figure 1. Dynamic expression of BRG1, BRM and Tβ4 in heart development and injury.

(**a**) qRT-PCR analysis of *Brg1*, *Brm* and *Tβ4* expression levels in developing (E12.5) and adult (intact and primed- (+Tβ4), injured-) hearts. (**b**) Immunoblotting studies using anti-

Wt1, anti-BRG1, anti-BRM and anti-GAPDH antibodies on protein extracts from E12.5 and adult (intact, primed- (+Tβ4) and non-primed MI) hearts. The original uncropped images of gels are shown in **Supplementary Fig. 12**. (**c-e**) Immunostaining for GFP (Wt1) and BRG1, BRM or Tβ4 in E12.5 hearts. Arrowheads mark epicardial cells co-expressing GFP and BRG1, BRM or Tβ4. (**f-h**) Immunostaining for GFP (Wt1) and BRG1, BRM or Tβ4 at day 4 post-MI (Adult, MI) showing co-localization in reactivated EPDCs (arrowheads). Note that even though injury alone led to expansion of the subepicardial space (indicated by double-arrows), *Wt1* (GFP) was weakly reactivated. All error bars are data ± s.d. Significant differences (P values) were calculated using two-tailed Student's t-test (*$P \leq 0.05$; **$P \leq 0.01$). ep, epicardium; my, myocardium; ses, subepicardial space. All scale bars 50μm, except **e**,**f** 100μm.

Vieira et al_Supplementary Figure 2

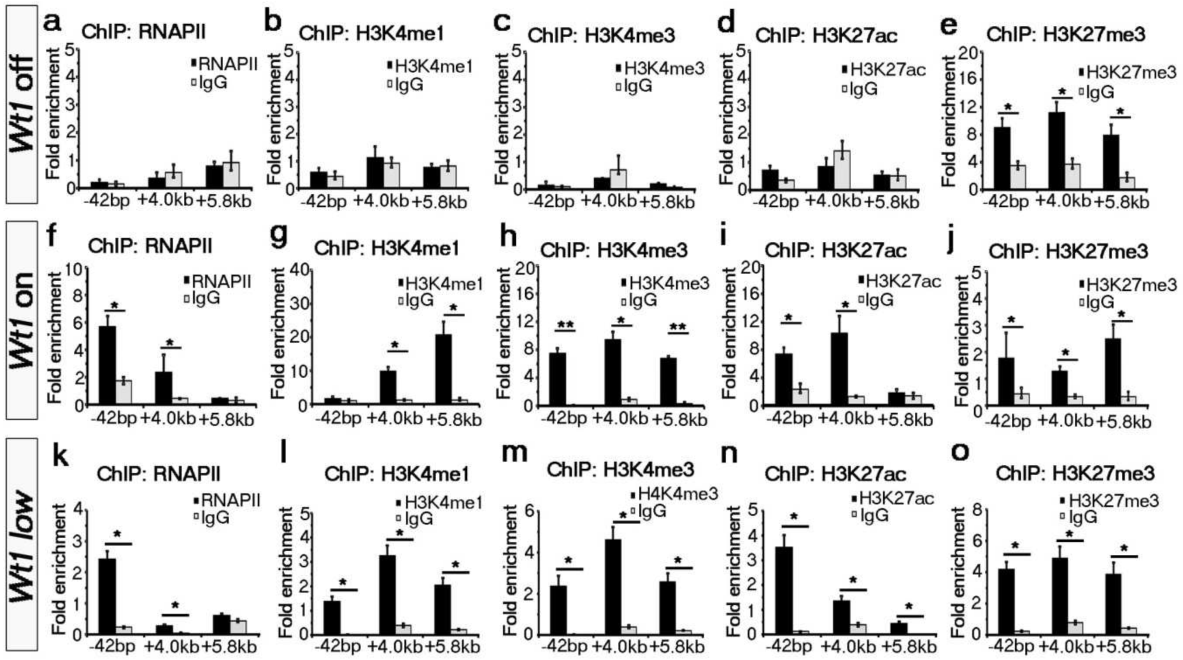

Supplementary Figure 2. Dynamic epigenetic regulation of *Wt1* ECRs in heart development and injury.

(**a-o**) Comparative RNA polymerase II-phosphorylated on serine 5 (RNAPII), H3K4me1, H3K4me3, H3K27ac and H3K27me3 ChIP-qPCR data from chromatin derived from hearts lacking (adult heart), exhibiting strong (embryonic and adult primed- (+Tβ4), post-MI hearts) or low *Wt1* activity (post-MI hearts). Three independent ChIP experiments per antibody per sample group (n = 3) were performed and are presented as fold enrichment over input. ChIP-qPCR data from chromatin derived from embryonic and adult primed- (+Tβ4), post-MI heart samples were combined and are shown in **f-j**. All error bars are data ± s.d. Significant differences (P values) were calculated using two-tailed Student's t-test (*$P \leq 0.05$; **$P \leq 0.01$).

Vieira et al_Supplementary Figure 3

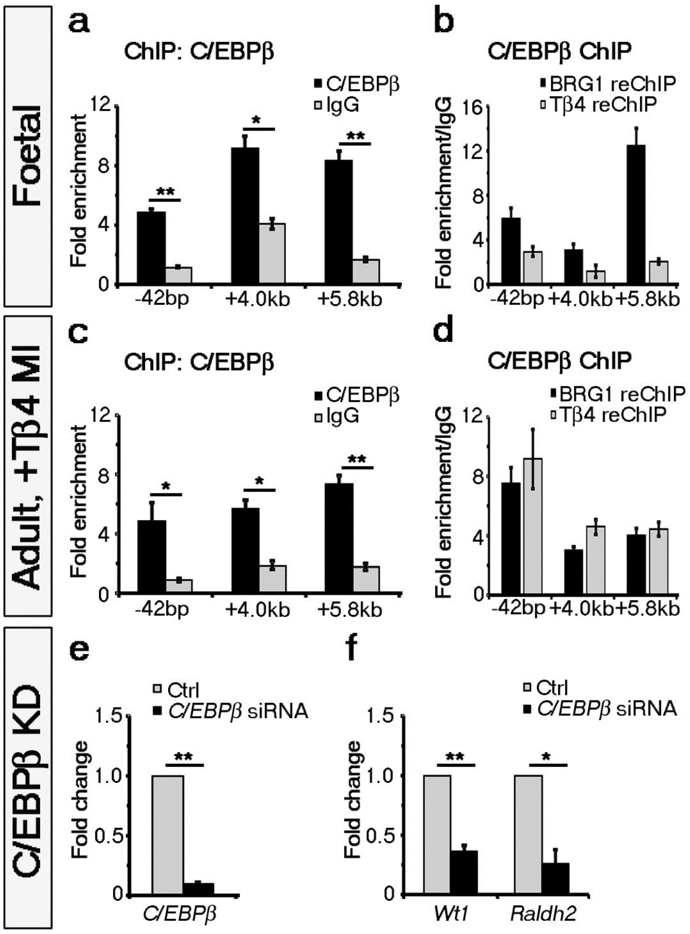

Supplementary Figure 3. The pioneer transcription factor C/EBPβ binds to *Wt1* ECRs and is required for *Wt1* expression.

(**a**) ChIP-qPCR data using chromatin derived from embryonic hearts at E11.5 and anti-C/EBPβ antibody. (**b**) Sequential ChIP (re-ChIP) with anti-BRG1 and anti-Tβ4 antibodies using embryonic heart-derived chromatin pulled-down with anti-C/EBPβ. (**c**) ChIP-qPCR data using chromatin derived from adult primed- (+Tβ4), injured-hearts at day 4 post-MI and an anti-C/EBPβ antibody. (**d**) re-ChIP with anti-BRG1 and anti-Tβ4 antibodies using C/EBPβ-enriched chromatin from Tβ4-primed post-MI hearts. (**e,f**) qRT-PCR analysis of *C/EBPβ*, *Wt1* and *Raldh2* transcript levels in mouse primary epicardial cells transfected with specific *C/EBPβ* siRNA sequences. Three independent experiments per antibody were performed using an average of 20 hearts at E11.5 and three adult hearts per experiment. ChIP

results are presented as fold enrichment over input, whereas re-ChIP results are present in fold enrichment over the level of ChIP with negative control IgG antibody. All error bars are data ± s.d. Significant differences (*P* values) were calculated using two-tailed Student's *t*-test (*$P \leq 0.05$; **$P \leq 0.01$).

Vieira et al_Supplementary Figure 4

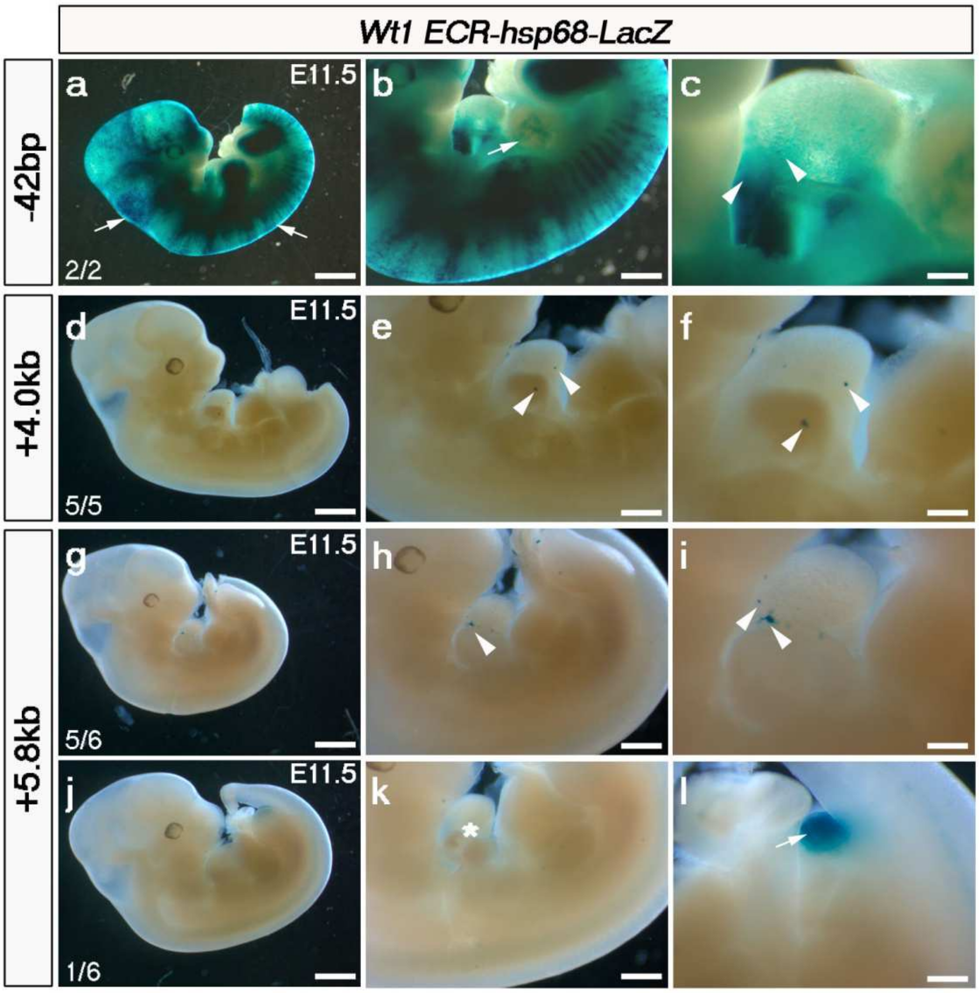

Supplementary Figure 4. ***Wt1*** **ECRs drive gene expression in the developing embryo.**

(**a-c**) -42bp ECR-driven *LacZ* expression (*n*=2 embryos) recapitulates *Wt1* pattern of expression in the developing embryo at E11.5, comprising domains in the spinal cord and brain (arrows in **a**), gut mesothelium (arrow in **b**) and epicardium (arrowheads in **c**). (**d-f**) +4.0kb ECR-driven *LacZ* expression (*n*=5 embryos) is restricted to the developing epicardium (arrowheads in **e** and **f**). (**g-l**) +5.8kb ECR-driven *LacZ* expression (*n*=6 embryos) is mostly restricted to the developing epicardium (5 out 6 embryos; arrowheads in **h** and **i**), but can also be detected in the urogenital region (1 out 6 embryos; arrow in **l**), where *Wt1* plays an essential role during development. Asterisk in **k** indicates lack of β-gal activity in the epicardium. 2-6 transgenic mice with independent ECR integration were examined to

assess the reproducibility of any given reporter activity pattern. Scale bars: **a,d,g,j** 1mm, **b,e,h,k** 500μm and **c,f,i,l** 160μm.

Vieira et al_Supplementary Figure 5

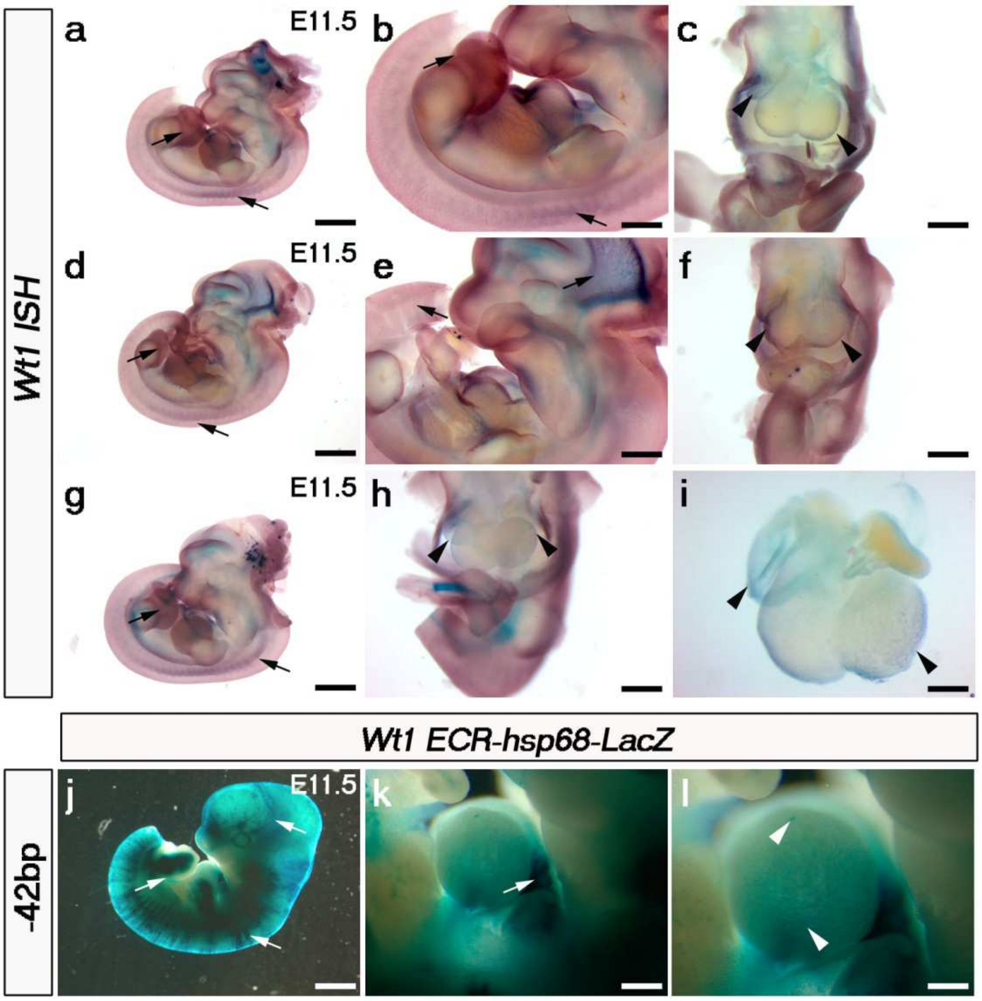

Supplementary Figure 5. The -42bp ECR recapitulates the pattern of expression of *Wt1* in the developing embryo.

(**a-i**) Whole-embryo *in situ* hybridization for *Wt1* mRNA in the developing embryo at E11.5, showing expression in the limb buds (arrows), head and spinal cord domains (arrows) and epicardial layer covering the ventricles and atria (arrowheads). (**j-l**) -42bp ECR-driven *LacZ* expression recapitulates *Wt1* pattern of expression in the developing embryo at E11.5, comprising domains in the spinal cord and brain (arrows) and epicardium covering ventricles and outflow tract (arrowheads). Similarities in the spatial pattern of expression of the *Wt1* mRNA and -42bp ECR-driven *LacZ* expression support the role of this ECR as the *Wt1* core promoter. Scale bars: **a,d,g,j** 1mm, **b,c,e,f,h,k** 500μm, **i** 160μm and **j** 100μm.

Vieira et al_Supplementary Figure 6

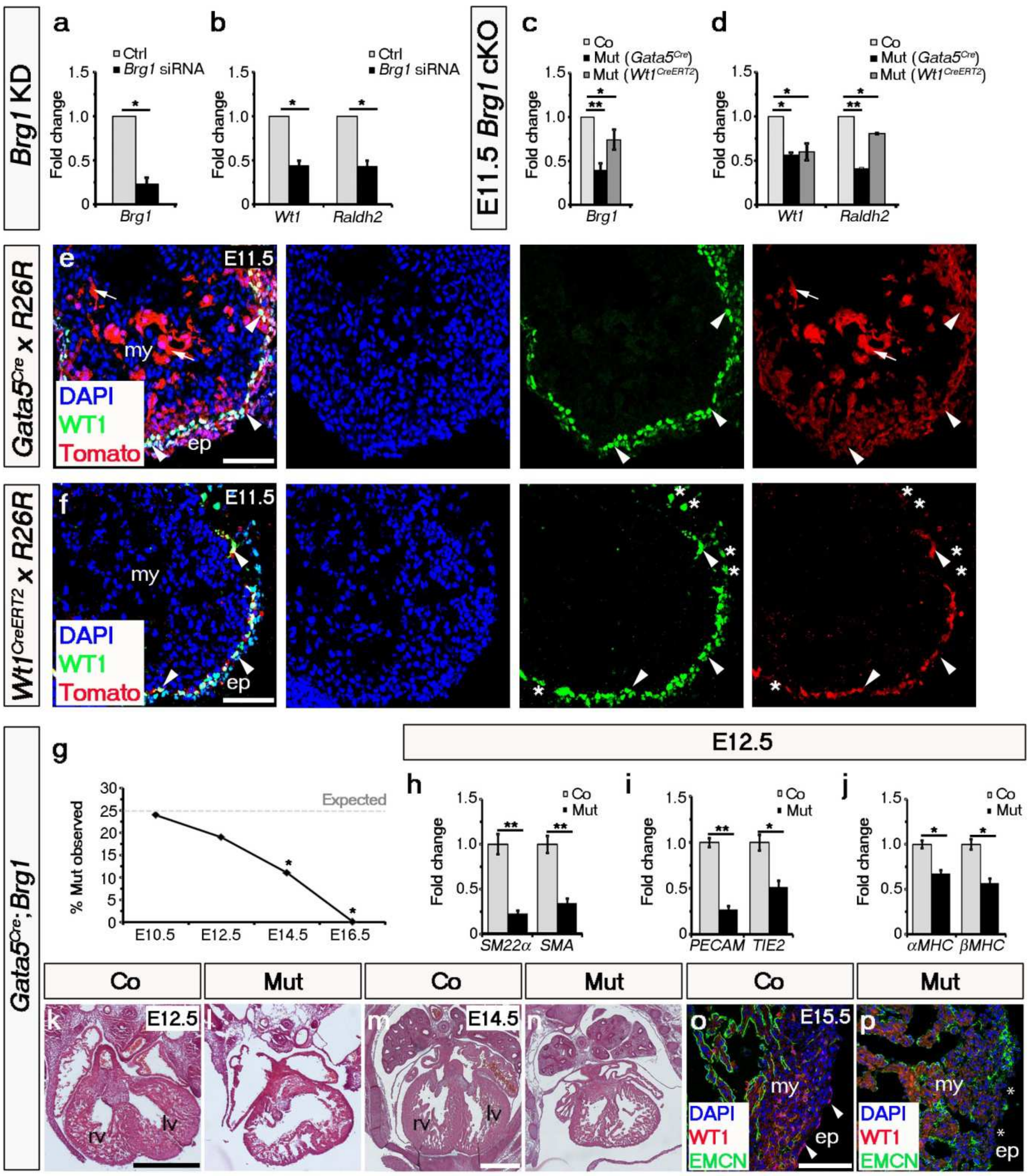

Supplementary Figure 6. BRG1 is required for *Wt1* expression, characterization of *Gata5*^{cre} and *Wt1*^{CreERT2} epicardial Cre lines, and phenotypic assessment of *Gata5*^{cre}*;Brg1*$^{F/F}$ mutant embryos.

(**a**,**b**) qRT-PCR analysis of *Brg1*, *Wt1* and *Raldh2* transcript levels in mouse primary epicardial cells transfected with specific *Brg1* siRNA sequences. (**c**,**d**) qRT-PCR analysis of

Brg1, *Wt1* and *Raldh2* expression levels in *Brg1* epicardial-deficient (cKO) embryonic hearts at E11.5 generated by crossing *Gata5^{Cre}* or *Wt1^{CreERT2}* strains with *Brg1$^{flox/flox}$* mice. (**e**) Native tomato fluorescence and Wt1 immunostaining analysis of coronal sections of embryonic *Gata5^{Cre};R26R^{tdTomato}* hearts at E11.5 documenting *Cre* activity domain extending beyond the Wt1-positive epicardium (arrowheads) including the developing myocardium and endocardium (arrows). (**f**) Native tomato fluorescence and Wt1 immunostaining analysis of coronal sections of embryonic *Wt1^{CreERT2};R26R^{tdTomato}* hearts at E11.5 showing that whilst *CreERT2* activity accurately targets the developing epicardium (arrowheads), targeting is not fully efficient as some Wt1-positive cells were tomato-negative (asterisks). (**g**) Percentage (%) of *Gata5^{cre};Brg1$^{F/F}$* mutant embryos observed in litters arising from crosses between a male of the genotype *Gata5^{Cre};Brg1$^{F/+}$* and a female of the genotype *Brg1$^{F/F}$* collected at E10.5 (24%; 8 mutants out of 33 embryos), E12.5 (19%; 13 mutant out of 69 embryos), E14.5 (11%; 8 mutants out of 73 embryos), E16.5 (0%; 0 mutants out of 23 embryos). (**h-j**) qRT-PCR analysis of *SM22α*, *αSMA*, *PECAM*, *Tie2*, α*MHC* and β*MHC* expression levels in *Gata5^{cre};Brg1$^{F/F}$* mutant (mut) and control (co; *Gata5^{Cre}* negative) embryonic hearts at E12.5. (**k-n**) Representative hematoxylin and eosin staining of transverse sections of E12.5 (**k,l**) and E14.5 (**m,n**) embryos arising from crosses between a male of the genotype *Gata5^{Cre};Brg1$^{F/+}$*and a female of the genotype *Brg1$^{F/F}$*. (**o,p**) Immunostaining for Wt1 and Endomucin (EMCN) in E15.5 control (co) and mutant hearts (mut; *Gata5^{cre};Brg1$^{F/F}$*; 1 mutant embryo out of 16 embryos). Arrowheads in **o** mark Wt1-expressing epicardial cells. Asterisks in **p** indicate lack of Wt1 expression in the epicardium. Note disorganized EMNC-expressing endocardium and lack of clear EMCN-positive blood vessel structures in the myocardium of mutant hearts. All error bars are data ± s.d. Significant differences (*P* values) in **a-d,h-j** were calculated using two-tailed Student's *t*-test, whilst a Chi-square test (χ^2) was performed in **g** to evaluate statistically significant differences between the observed and

expected (25%; grey line in **g**) numbers of *Gata5*^{cre};*Brg1*$^{F/F}$ mutant embryos, assuming Mendelian inherence of a single copy number of *Cre* (*$P \leq 0.05$; **$P \leq 0.01$). co, control; ctrl, control; ep, epicardium; lv, left ventricle; mut, mutant; my, myocardium; rv, right ventricle. All scale bars 100µm, except **k-n** 1mm.

Vieira et al_Supplementary Figure 7

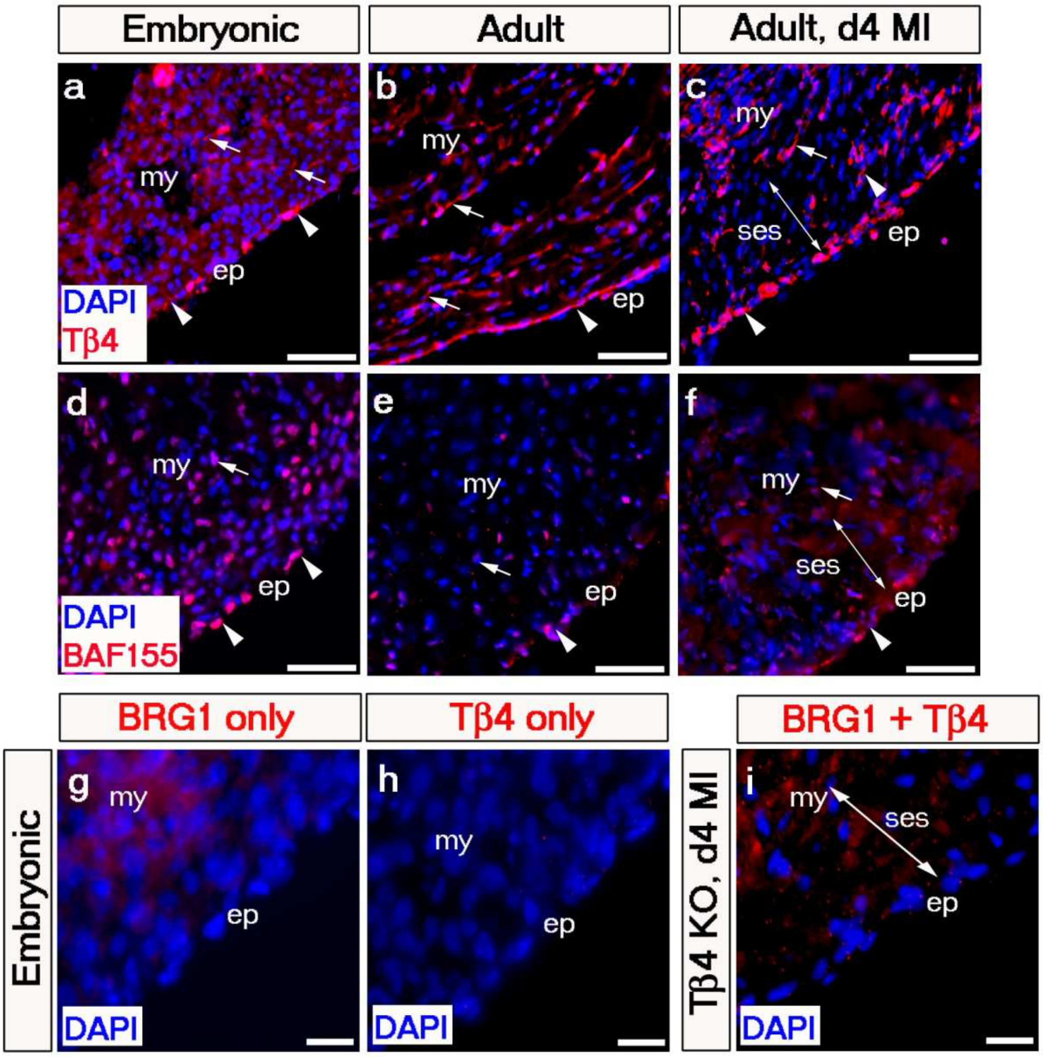

Supplementary Figure 7. Characterization of Tβ4 and BAF155 expression in heart development and injury.

(**a-c**) Immunostaining for Tβ4 in embryonic, adult-intact and adult-post-MI hearts. (**d-f**) Immunostaining for BAF155 in embryonic, adult-intact and adult-post-MI hearts. Arrows mark the myocardium, arrowheads mark the epicardium and double-arrow indicates the extended subepicardial space. (**g,h**) Duolink in situ analysis using PLA probes specific for BRG1 or Tβ4 alone revealed no nuclear PLA red signals (speckles) in the developing myocardium or epicardium. (**i**) Combination of PLA probes against anti-BRG1 and anti-Tβ4 antibodies revealed no nuclear speckles in the myocardium, epicardium or subepicardial

space of post-MI hearts from *Tβ4* knockout mice. ep, epicardium; my, myocardium; ses, subepicardial space. All scale bars 100 μm, except **g-i** 50 μm.

Vieira et al_Supplementary Figure 8

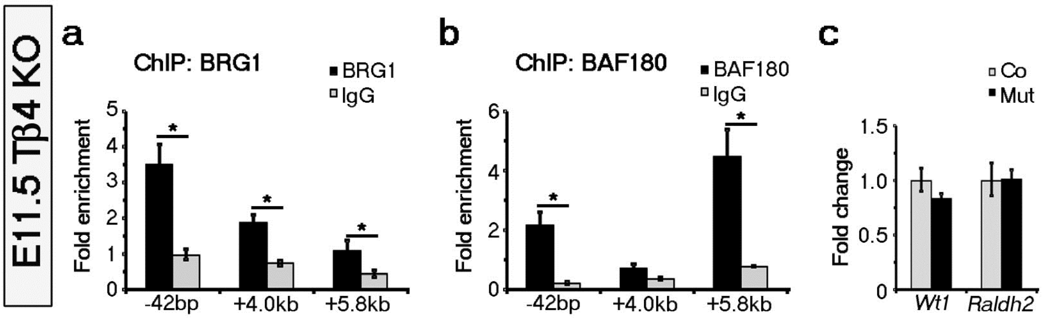

Supplementary Figure 8. Endogenous Tβ4 is not required for SWI/SNF-mediated expression of *Wt1* in the developing heart.

(**a,b**) Profiling of Brg1 and BAF180 occupancy within the *Wt1* locus by ChIP-qPCR using chromatin derived from hearts of *Tβ4*-deficient embryos. (**c**) qRT-PCR analysis documenting no significant differences in *Wt1* or *Raldh2* (downstream target of *Wt1*) expression in hearts from *Tβ4*-deficient embryos comparing to wild-type littermates. Three independent ChIP experiments per antibody per sample group (n = 10 fetal hearts) were performed and are presented as fold enrichment over input. All error bars are data ± s.d. Significant differences (P values) were calculated using two-tailed Student's t-test ($*P \leq 0.05$).

Vieira et al_Supplementary Figure 9

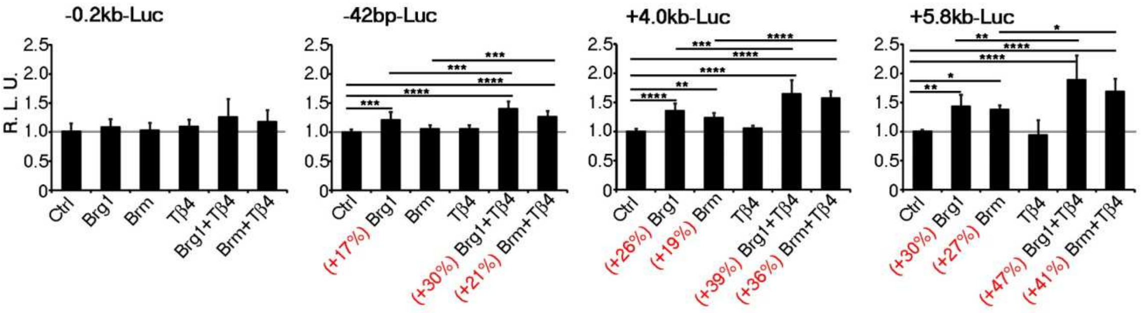

Supplementary Figure 9. Tβ4 augments the activation of *Wt1* ECRs by SWI/SNF.

Examination of transcriptional activity of -0.2kb, -42bp, +4.0kb and +5.8kb ECRs by luciferase reporter assays in SW13 cells upon restoration of *Brg1* or *Brm* alone or in combination with Tβ4 nuclear overexpression. Three independent experiments with each experimental condition done in triplicated were performed. The increase in the transcriptional activity (%) between each experimental condition and control is highlighted in red. Please note that these reporter assays are heterologous system, utilising episomal vectors, as opposed to studying naked plasmid DNA. Most luciferase reporter assays focus on the latter, and the expectation is to observe significant fold-changes in expression with addition of the factors being tested. Here the effect of chromatin-remodelling on *Wt1* ECRs is probed and the system is at the limit of detection given the need for synthesis/packaging of chromatin-like DNA (via the episomal vectors) and appropriate expression levels of the reporters (firefly luciferase in pREP4 and TK-*Renilla* luciferase in pREP7), remodellers (Brg1 or Brm) and co-factors (Tβ4). Despite these technical limitations, relative increases with addition of Brg1/Brm and Tβ4 combined were observed, as compared to Brg1/Brm alone across the three ECRs, providing confidence-in-the-data as it stands. All error bars are data ± s.d. Significant differences (*P* values) were calculated with one-way ANOVA followed up by the

Tukey multiple comparison test (*$P \leq 0.05$; **$P \leq 0.01$; ***$P \leq 0.001$; ****$P \leq 0.0001$). r.l.u., relative luciferase unit, after normalization to Renilla.

Vieira et al_Supplementary Figure 10

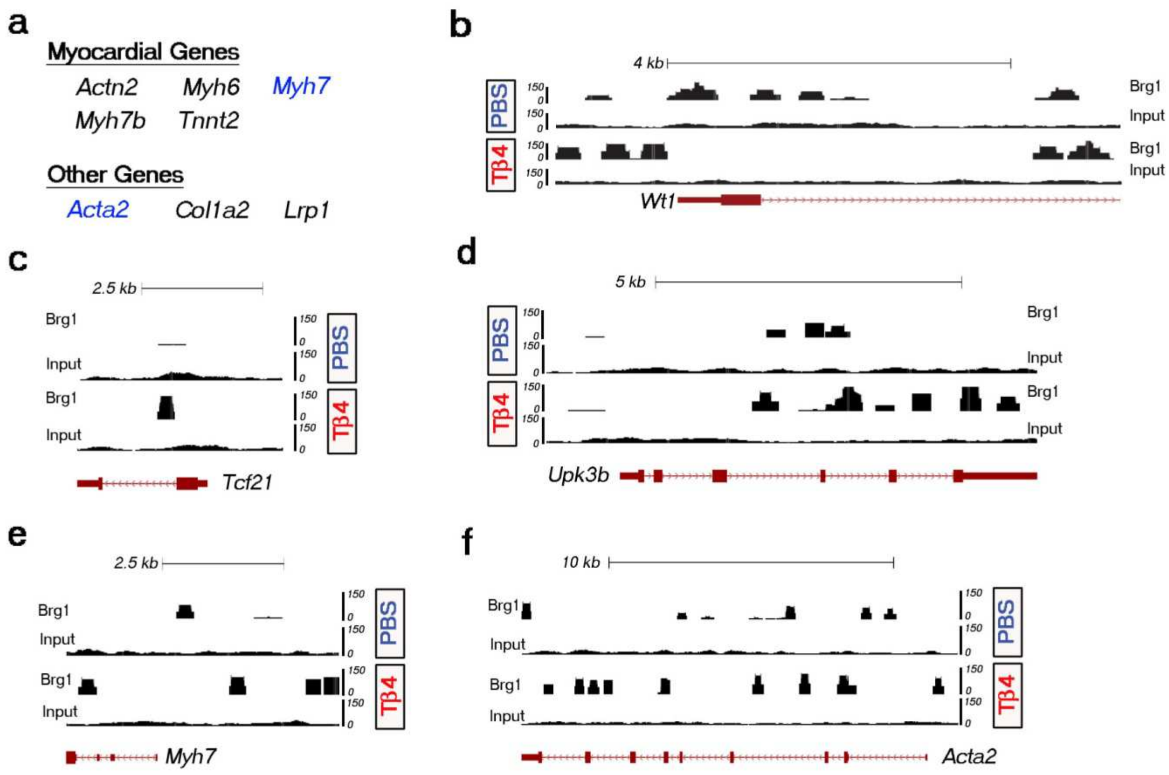

Supplementary Figure 10. Genome-wide BRG1-enriched regions.

(**a**) List of representative myocardial and other BRG1-enriched genes. (**b-f**) Representative UCSC browser snapshots of selected loci, derived from the Brg1 ChIP-seq experiments, showing comparatively more peaks in the Tβ4-primed, -injured adult heart samples, compared to the non-primed (PBS), -injured adult heart samples. Please note that *zoomed-in* views are shown for the *Wt1* (**b**) and *Myh7* (**e**) loci in order to highlight previously described BRG1-bound regulatory elements located within 5kb of the transcriptional start site (*Myh7*[1]) or intron 1 (*Wt1*; this study).

Vieira et al_Supplementary Figure 11

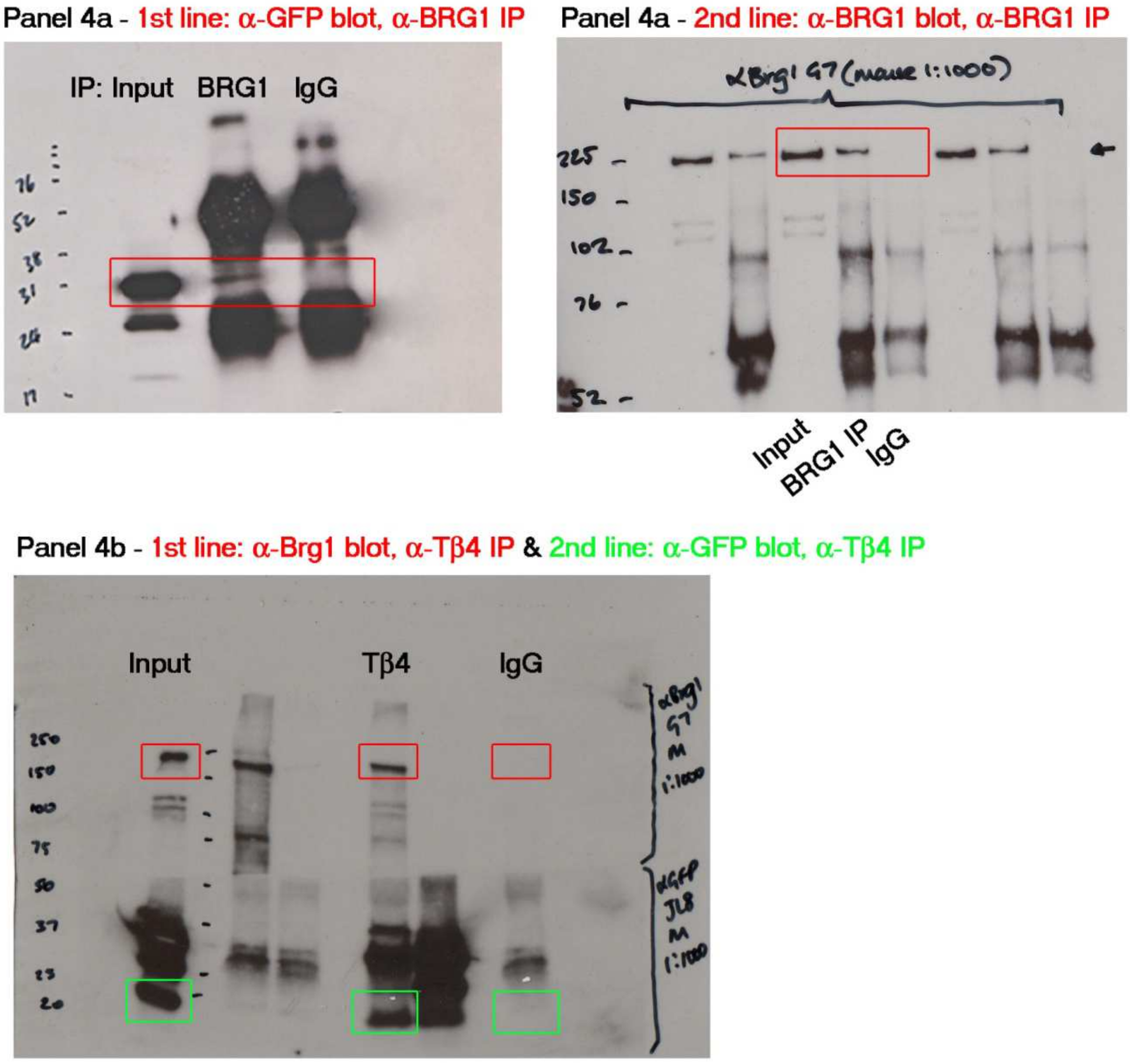

Supplementary Figure 11. Full unedited gels used in Figure 4, panels a and b (co-IP).

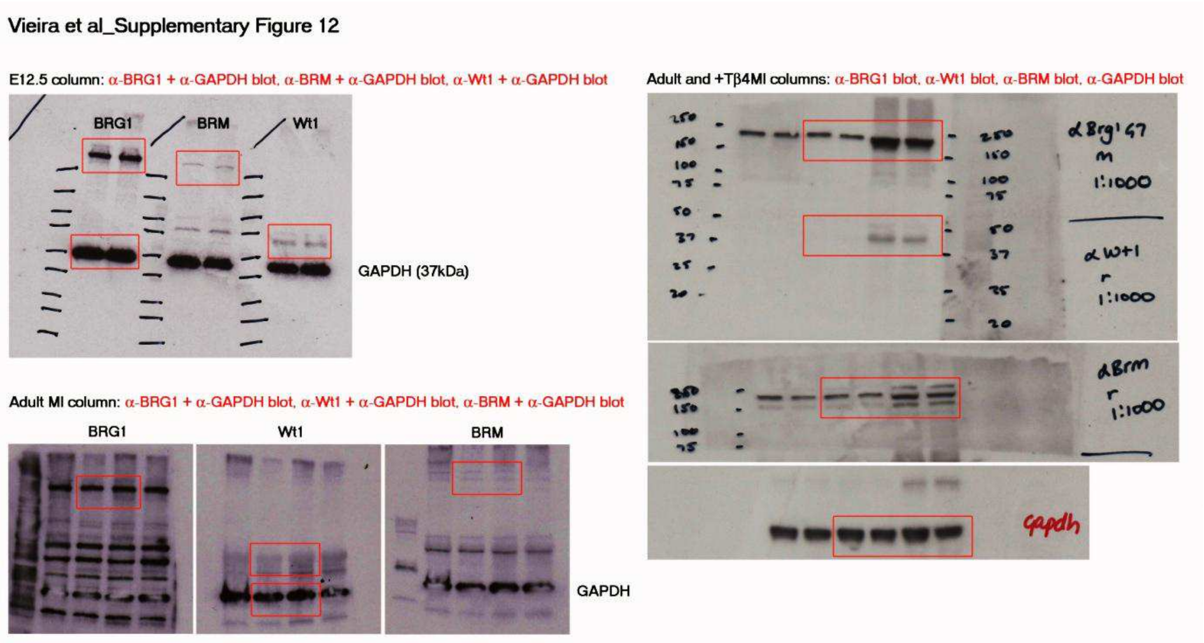

Supplementary Figure 12. Full unedited gels used in Supplementary Figure 1, panel b (western blotting).

Supplementary Table 1. Evolutionary conserved regions (ECRs) within *Wt1* locus

ECR	Location	% Homology (vs. human)	Size (bp)
-4.4kb	chr2:105122151-105122543	87.5	393
-3.4kb	chr2:105123127-105123252	80.2	126
-0.3kb	chr2:105126192-105126292	80.2	101
-0.2kb	chr2:105126355-105126483	79.8	129
-42bp	chr2:105126487-105126925	86.3	439
+4.0kb	chr2:105130624-105130729	81.1	106
+5.8kb	chr2:105132363-105132550	78.7	188

Supplementary Table 2. Predicted C/EBPβ binding sites in *Wt1* ECRs by TRANSFAC

ECR	# Sites	Sequence	Location/ Strand	Score
-42bp	1	agctttgGGAAGct	109 (+)	95.0
	2	agctgggGTAAGga	137 (+)	96.5
+4.0Kb	1	accgtggGAAAGtg	35 (+)	98.1
+5.8Kb	1	aattaatGAAAGt	90 (+)	80.7

Supplementary Table 3. List of primers and applications

**ChIP-qPCR**		
**ECR**	**Forward (5’-3’)**	**Reverse (5’-3’)**
-4.4kb	TTCTCCTCCTCCTCCTCCTC	AGGGGCTAAAACCACCTAGC
-3.4kb	GCAAGGCTACAGCGTGTTTA	GGCGCATAATTATTACAAGATGAA
-0.3kb	CCCCAAAGTTAGGCTATCTGC	TAATGAGTCCCCTCGGTGTC
-0.2kb	GACACCGAGGGGACTCATTA	CCCTAGCCTAGCTCAGCAAA
-42bp	ACACCCCCGGTGCTAGTAA	CAGCTTCCCAAAGCTCAAA
+4.0kb	GGAGGAGAGCTCAGAGCCTTA	GCAGAGAGATTGCTGACTTCG
+5.8kb	CCCCCTACAAGCTTTCCTAAA	ACAAACAACACCGTGGCTCT
(-)ECR	CTGGAAACTGAGCCCTATGC	TGTAGCCTTGCGATCTGTCA
***Real time* qRT-PCR**		
**Gene**	**Forward (5’-3’)**	**Reverse (5’-3’)**
Wt1	TTCAAGGACTGCGAGAGAAG	GGGAAAACTTTCGCTGACAA
Raldh2	TGAGTTTTGGCTTACGGGAGT	TTGTTGTGAGGCAAGAGTGG
Brg1	CAAAGACAAGCATATCCTAGCCA	CACGTAGTGTGTTTAAGGACC
Brm	CTCCTGGACCAATTCTGGGG	CATCGTTGACAGAGGATGTGAG
Tβ4	ATGTCTGACAAACCCGATATGGC	CCAGCTTGCTTCTCTTGTTCA
C/EBPβ	GGCCCGGCTAGACAGTTAC	GTTTCGGGACTTGATGCAAT
SM22α	CAACAAGGGTCCATCCTACGG	ATCTGGGCGGCCTACATCA
SMA	GTCCCAGACATCAGGGAGTAA	TCGGATACTTCAGCGTCAGGA
PECAM	CTGCCAGTCCGAAAATGGAAC	CTTCATCCACCGGGGCTATC
Tie2	CGGCCAGGTACATAGGAGGAA	TCACATCTCCGAACAATCAGC
αMHC	GCCCAGTACCTCCGAAAGTC	GCCTTAACATACTCCTCCTTGTC
βMHC	ACTGTCAACACTAAGAGGGTCA	TTGGATGATTTGATCTTCCAGGG
Hprt	TCAGTCAACGGGGGACATAAA	GGGGCTGTACTGCTTAACCAG
18S	GCCGCTAGAGGTGAAATTCTTG	GAAAACATTCTTGGCAAATGCTTT

Supplementary Table 4. List of antibodies and applications

**Immunofluorescence**		
**Antibody**	**Company**	**Dilution**
Rabbit anti-Tβ4	Immunodiagnostik	1:100
Rabbit anti-Brg1	Abcam epitomics	1:200
Rabbit anti-Brm	Abcam	1:50
Rabbit anti-Baf155	Santa Cruz	1:50
Chicken anti-GFP	Abcam	1:1000
Rabbit anti-Wt1	Abcam	1:200
Rat anti-Endomucin (V.7C7)	Santa Cruz	1:100
Rabbit anti-SM-MHC11	Abcam	1:100
**IP and western blotting**		
**Antibody**	**Company**	**Dilution**
Mouse anti-GFP (JL8)	Clontech	1:1000
Mouse anti-Brg1 (G7)	Santa Cruz	1:1000
Rabbit anti-Tβ4 (FL-44)	Santa Cruz	1:1000
Rabbit anti-Brm	Abcam	1:1000
Rabbit anti-Wt1	Abcam	1:1000
Mouse anti-GAPDH	Millipore	1:1000
**Duolink**		
**Antibody**	**Company**	**Dilution**
Rabbit anti-Tβ4	Immunodiagnostik	1:100
Mouse anti-Brg1 (G7)	Santa Cruz	1:50
Rabbit anti-Baf155	Santa Cruz	1:50
**ChIP (all ChIP grade)**		
**Antibody**	**Company**	
Rabbit anti-Brg1	Millipore	5μg
Rabbit anti-Brm	Abcam	5μg
Rabbit anti-Baf180	Millipore	5μg

**ChIP (all ChIP grade)**		
**Antibody**	**Company**	
Rabbit anti-Tβ4 (FL-44)	Santa Cruz	5μg
Rabbit anti-RNAPII phospho S5	Abcam	5μg
Rabbit anti-H3K4me1	Abcam	5μg
Rabbit anti-H3K4me3	Abcam	5μg
Rabbit anti-H3K27ac	Abcam	5μg
Rabbit anti-H3K27me3	Abcam	5μg

Supplementary References

1. Hang,C.T. *et al.* Chromatin regulation by Brg1 underlies heart muscle development and disease. *Nature* **466**, 62-67 (2010).
